# Supplementary material for: Nanoscale tomography reveals the deactivation of automotive copper-exchanged zeolite catalysts
Source: Nat Commun. 2017 Nov 21;8:1666. doi: 10.1038/s41467-017-01765-0 (PMC5698465; doi:10.1038/s41467-017-01765-0)
Supplement: Supplementary file 1 — Supplementary Information [file 41467_2017_1765_MOESM1_ESM.pdf]

Supplementary Methods

Powder X-ray diffraction patterns

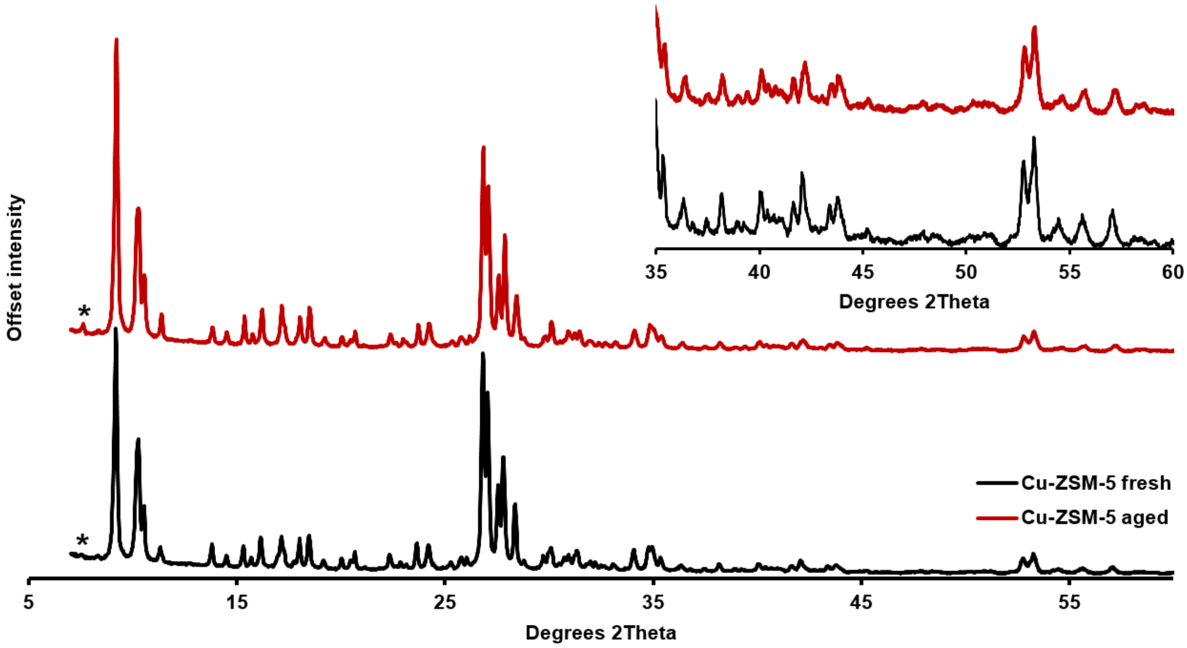

Supplementary Figure 1. **X-ray diffraction patterns of fresh and aged Cu-ZSM-5.** In the inset image, the intensity has been increased by a factor of 10 for clarity. The * indicates the Co k_β peaks.

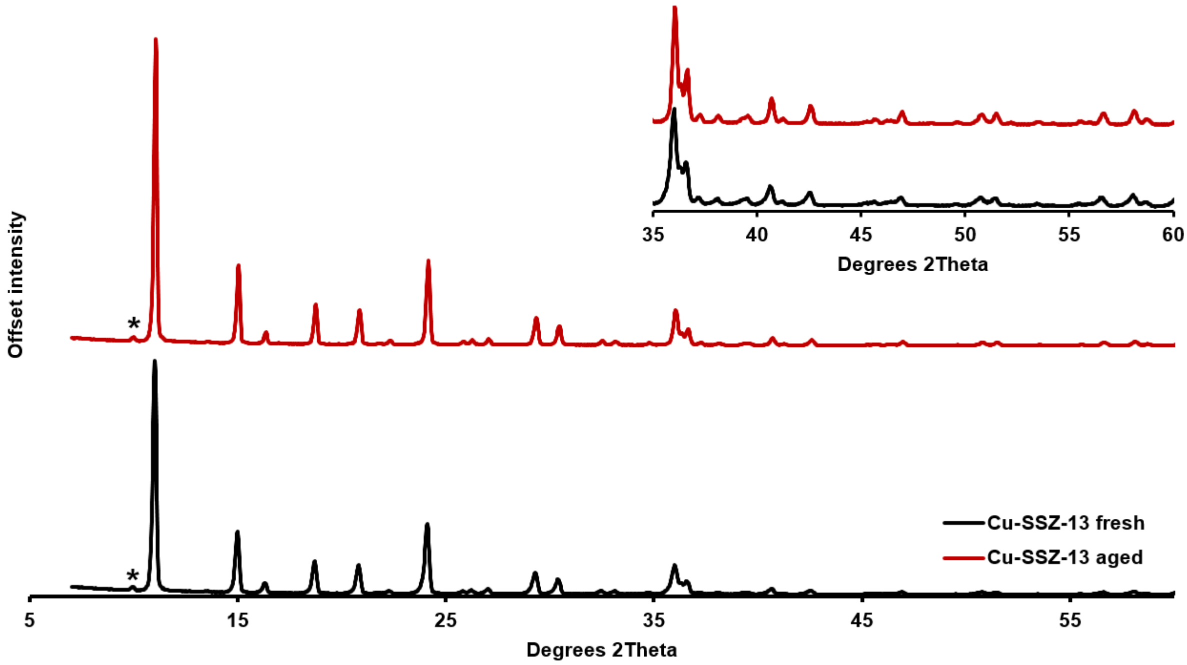

Supplementary Figure 2. X-ray diffraction patterns of fresh and aged Cu-SSZ-13. In the inset image, the intensity has been increased by a factor of 10 for clarity. The * indicates the Co k_β peak.

UV–Vis-NIR DRS spectra

The spectra of the Cu-exchanged zeolite samples are in Supplementary Figure 3 and the spectra of reference materials are in Supplementary Figure 4. All hydrated, Cu-exchanged zeolite samples contain two sharp features at 7140 and 5260 cm^{-1}, which are due the overtone and combination bands of physisorbed water[1]. These bands disappear upon dehydration, demonstrating that the samples are well-dehydrated after calcination. In hydrated Cu-SSZ-13 the adsorption bands near 12,500 cm^{-1} correspond to d-d transitions in Cu^{2+} species. These species are also present in hydrated Cu-ZSM-5 and in dehydrated Cu-ZSM-5, although with a slight blue shift[1–3]. In dehydrated Cu-SSZ-13 it is well known that these d-d transitions split into four bands as removal of the water allows Cu^{2+} to interact more strongly with the framework and be stabilized in a more distorted local environment. It is known that this only occurs in zeolite SSZ-13 due to its highly distorted environment, and not in zeolite ZSM-5[4]. Upon aging, there is a change in the d-d transition region in zeolite Cu-SSZ-13 and we believe this is due to Cu migration to different framework positions. In all hydrated Cu-exchanged zeolite samples there is a ligand to metal charge transfer (LMCT) band near 48,000 cm^{-1} associated with ($O^{2-}Cu^{2+} \rightarrow O^{-}Cu^{+}$). In dehydrated Cu-exchanged zeolite samples it is known that there is a red shift in the LMCT band due to changes in the local environment of Cu^{2+}, which can also occur with aging, and in zeolite Cu-SSZ-13 the main shift is reported at 41,500 cm^{-1} with shoulders at 32,000 and 29,000 cm^{-1}, while in zeolite Cu-ZSM-5 there is a red shift to 41,000 cm^{-1} [1]. Spectroscopic features associated with the presence of CuO species appear in the 35,000-40,0000 cm^{-1} range, and are present in aged zeolite Cu-ZSM-5, and to a lesser extent in zeolite Cu-SSZ-13[2,3,5]. Reference spectra of CuO and $CuAl_2O_4$ spinel are included in Supplementary Figure 4. In the dehydrated, oxygen activated Cu-exchanged zeolite samples $[Cu\text{-}O\text{-}Cu]^{2+}$ dimers are reported at 22,700 cm^{-1}, but we do not see this in our samples, excluding their presence[1].

It is important to mention here that the reference spectra of $CuAl_2O_4$ spinel and CuO were taken after mixing each species to 6 weight % with Na-form ZSM-5 (CBV2314 was exchanged to Na-form using 1 M NaCl solution, full exchange was followed by pH and the zeolite was subsequently calcined) to dilute the species and prevent detector saturation. The sodium form of the zeolite was used to prevent Cu exchange. $CuAl_2O_4$ spinel was prepared following reference [6], while CuO was prepared by heating $Cu(OH)_2$ in basic solution to ~80°C until a black solid was obtained. The difference spectra are also presented to remove the influence of the zeolite.

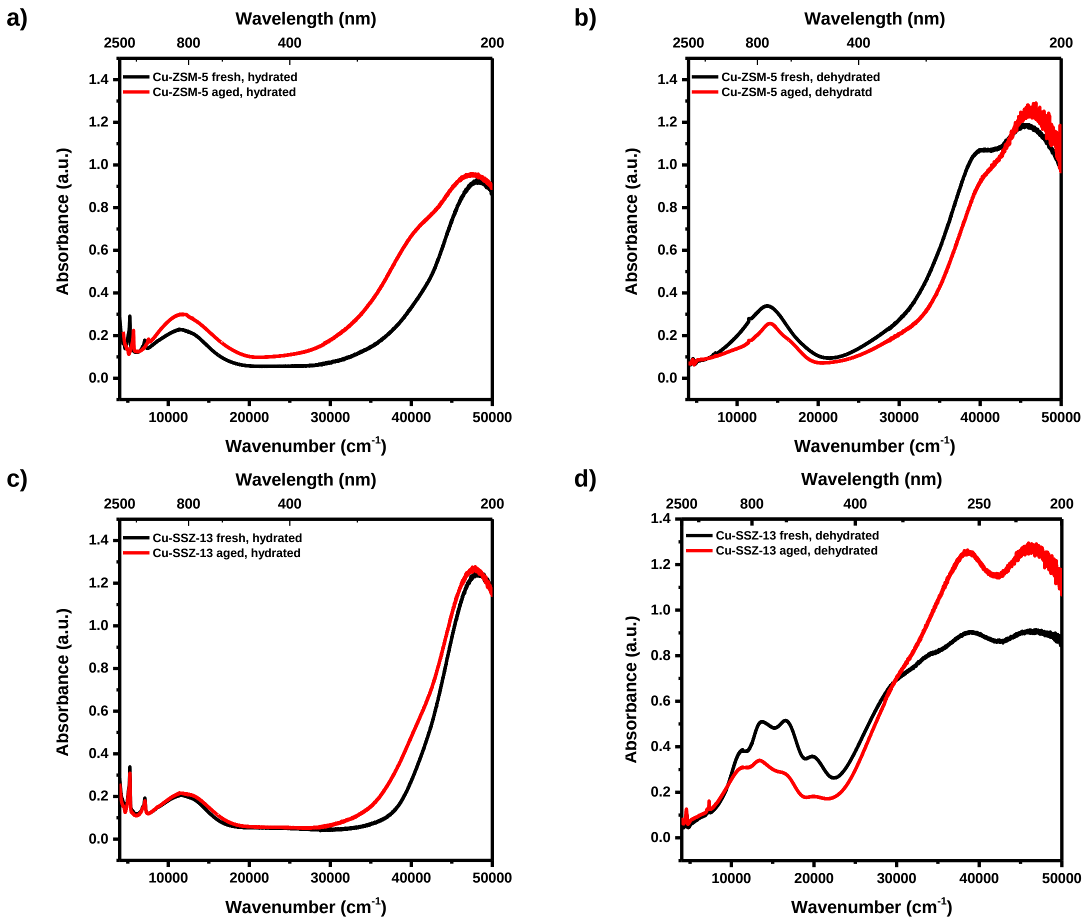

Supplementary Figure 3. UV–Vis-NIR DRS spectra of all materials. a, Hydrated, fresh and aged Cu-ZSM-5. **b,** Dehydrated, fresh and aged Cu-ZSM-5. **c,** Hydrated, fresh and aged Cu-SSZ-13. **d,** Dehydrated, fresh and aged Cu-SSZ-13.

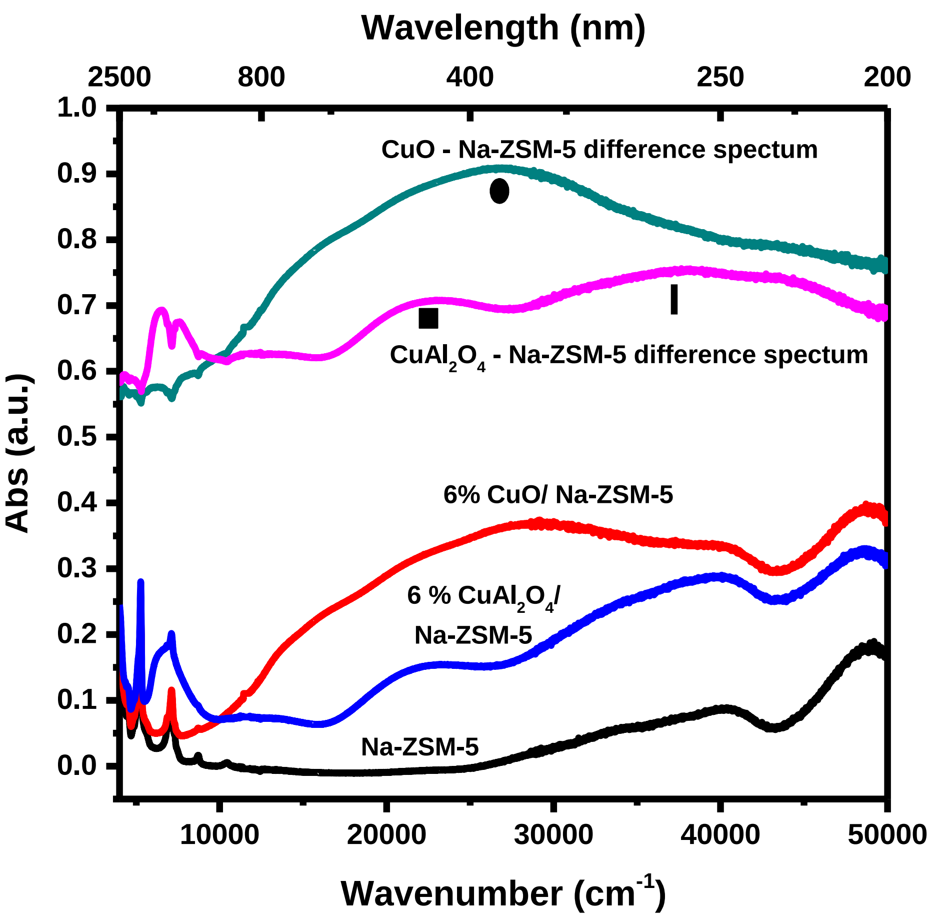

Supplementary Figure 4. UV–Vis-NIR DRS spectra of Na-ZSM-5, 6% $CuAl_2O_4$ spinel in Na-ZSM-5, 6% CuO in Na-ZSM-5, as well as the difference spectra after subtracting the Na-ZSM-5 spectra to reveal the spectroscopic features of interest.

Ammonia temperature programmed desorption

Strong, intermediate and weak acid sites were characterized using NH_3-TPD, and the results are shown in Supplementary Figure 5. Strong acid sites are Brønsted acidic framework Al, intermediate strength sites are from exchanged Cu, and weak sites are due to framework defects and extraframework Al[7]. Changes with aging are observed in both catalysts, with a decrease in strong acid sites attributed to framework Al removal, and Cu ions are identified in all samples as intermediate strength sites[3].

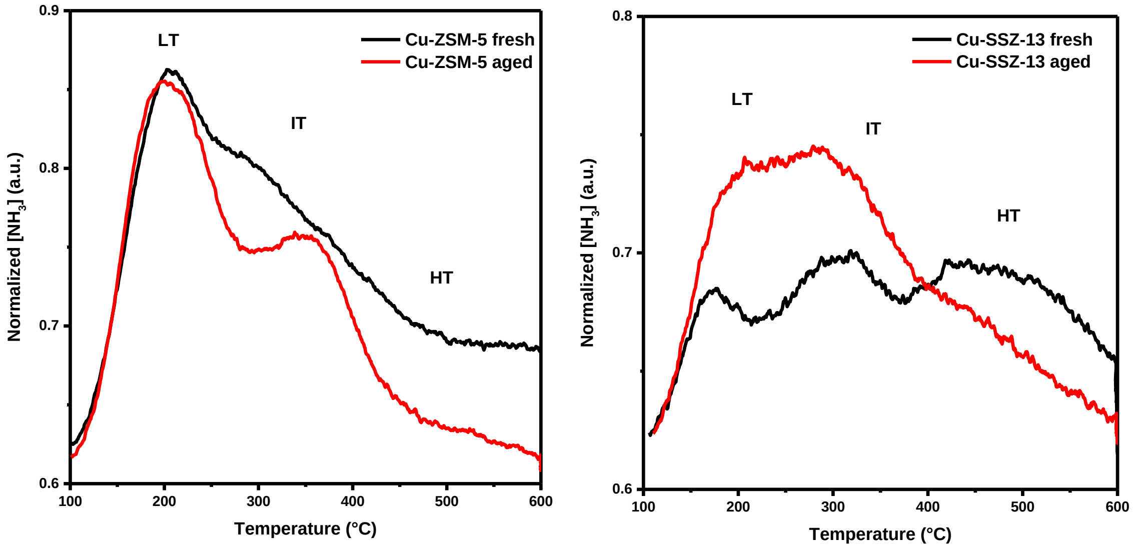

Supplementary Figure 5. Temperature programmed desorption (TPD) of NH_3. Results for fresh and aged zeolite Cu-ZSM-5 (left) and zeolite Cu-SSZ-13 (right). LT low temperature, HT high temperature, IT intermediate temperature ranges are indicated. The LT peak is normally assigned to Lewis acid sites inside the zeolite framework and the HT peak is assigned to Brønsted acid sites. The IT peak is due to interaction of Cu^{2+} with NH_3.

SEM characterization

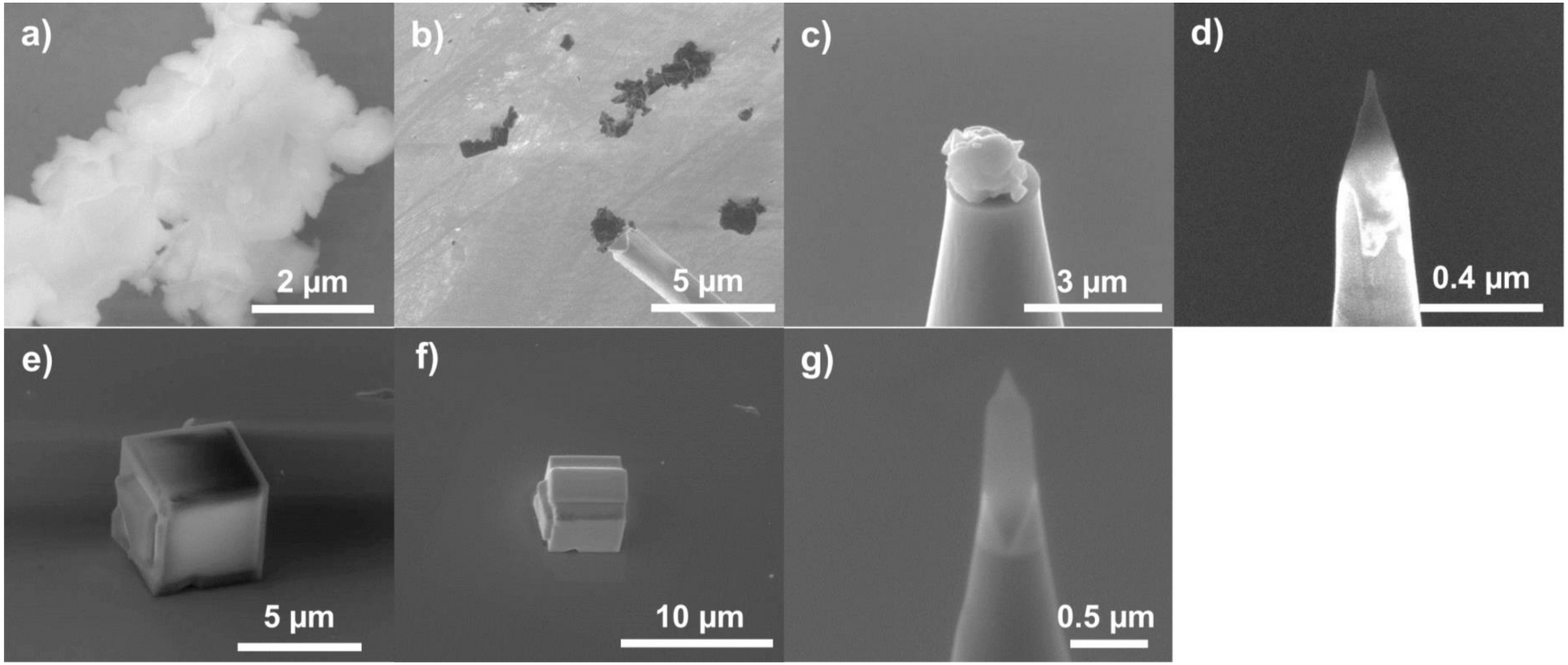

Supplementary Figure 6. Scanning electron microscopy (SEM) images of catalysts and needles for APT experiment. a, Crystals of fresh Cu-ZSM-5. **b,** Crystals of fresh Cu-ZSM-5 being transferred using electrostatic attraction. **c,** Fresh Cu-ZSM-5 attached prior to FIB milling into APT needle. **d,** Needle of fresh Cu-ZSM-5 prior to APT analysis. **e,** Fresh Cu-SSZ-13. **f,** Fresh Cu-SSZ-13 with a Pt strap deposited on the crystal prior to FIB cutting. **g,** Needle of fresh Cu-SSZ-13 prior to APT analysis.

APT data analysis

All error analyses were calculated from counting statistics using the method described in reference [8]. In all subsequent discussions, consistent with the terminology used in APT work, 'bulk' refers to the entire needle, 'matrix' refers to the atoms that remain after clusters are removed, and solute refers to the element(s) of interest in cluster analysis. See reference [9] for further details and definitions. In all APT analyses, a small amount of gallium was found to be present due to the FIB-cutting process, and our treatment of this potential influence is discussed in the subsection "Assessing Ga damage." All compositional percentages referred to in this work are atomic percentages. The non-conductive nature of the materials used in this study complicates the data collection. The pulsed laser heating helps to overcome these difficulties. Sample heating creates issues with processing the sample mass spectra as it creates thermal tails in the data. Due to this issue, not all collected data sets gave reliable, quantitative results, and only data sets that gave reliable results are presented. It is known that there are difficulties in quantitatively detecting oxygen or oxides as these tend to field evaporate as double detector hits, which will be registered as a single hit, and there is also evidence that molecular ions can dissociate after field evaporation, such that one ion becomes deionized and does not hit the detector within the appropriate time-of-flight window and is registered as background (even though we attempted to keep the detection rate low, 1 ion per 200 pulses)[9–11]. These issues can cause deviations from the actual composition, and hard to control experimental parameters can influence the severity of these artefacts. Therefore, we have concluded that APT has difficulty in quantifying the Al content in zeolite materials and we emphasized trends (isosurfaces, clustering, radial distribution function, etc.) rather than comparing absolute numbers. The Local Range-Assisted background estimate embedded in IVAS was used, which estimates the background based on the number of counts before and after the ranged peak.

TOF-MS analysis
Data from the APT TOF-MS for each material is shown below (samples 1, 3, 4 and 6) in Supplementary Figure 7 to Supplementary Figure 14 (bin width 0.03 Da), along with the peak assignments. For each sample two different spectra are included to demonstrate the change after exclusion of the Ga rich region from the data analysis. All peaks were ranged in the same location between samples, but the width varied based on the full width at half maximum (FWHM) of the peak and the thermal tails. The peaks were ranged to approximately two times the background unless another peak was present before this occurred. SEM images of samples 1, 3, 4 and 6 prior to the APT experiment are shown in Supplementary Figure 15 to illustrate differences in the tip shapes which may account for differences in the TOF-MS data.

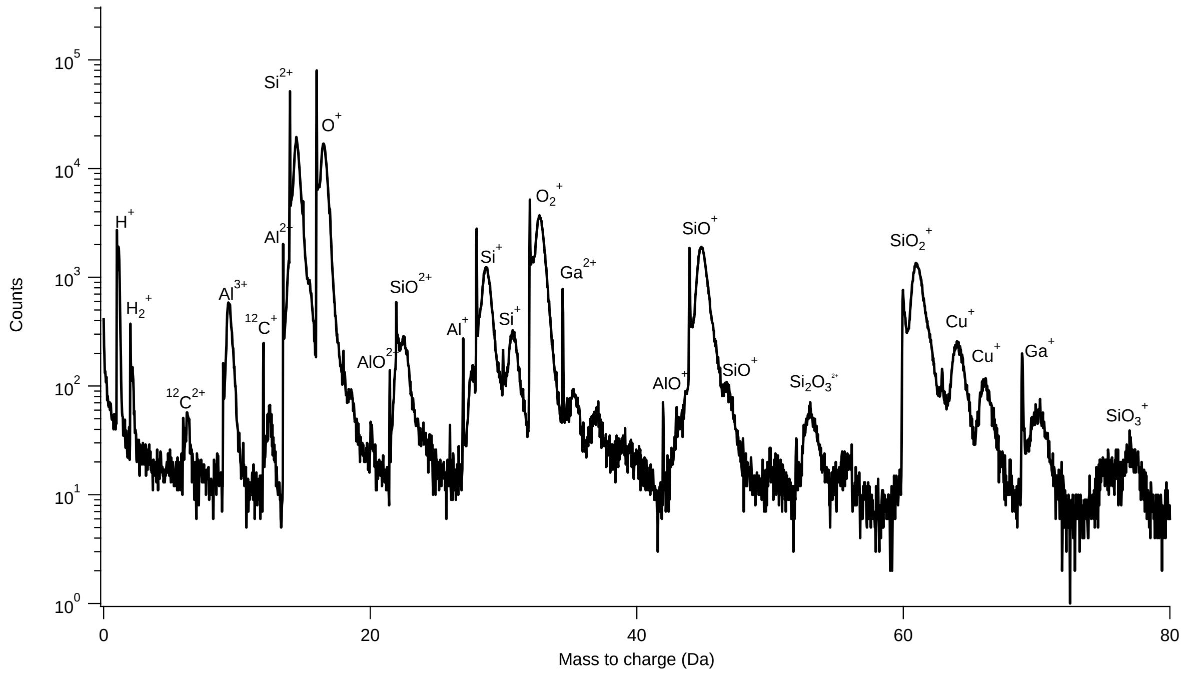

Supplementary Figure 7. TOF-MS data scale for fresh Cu-ZSM-5 (sample 1) before exclusion of Ga rich region.

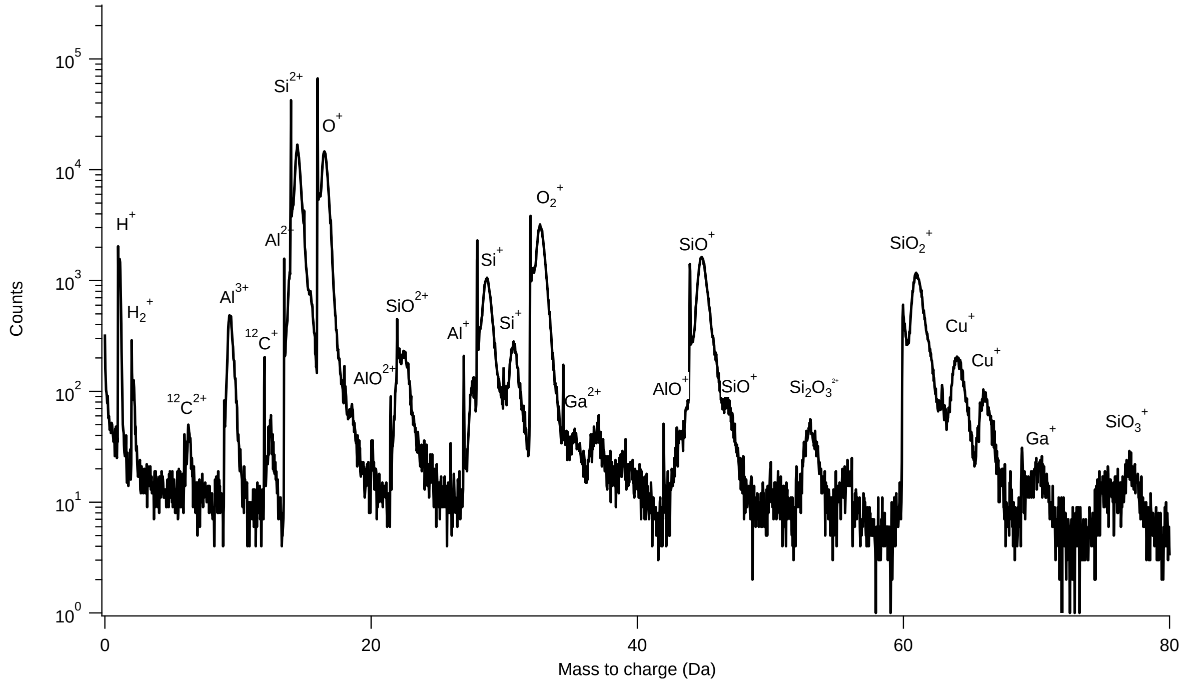

Supplementary Figure 8. TOF-MS data scale for fresh Cu-ZSM-5 (sample 1) after exclusion of Ga rich region.

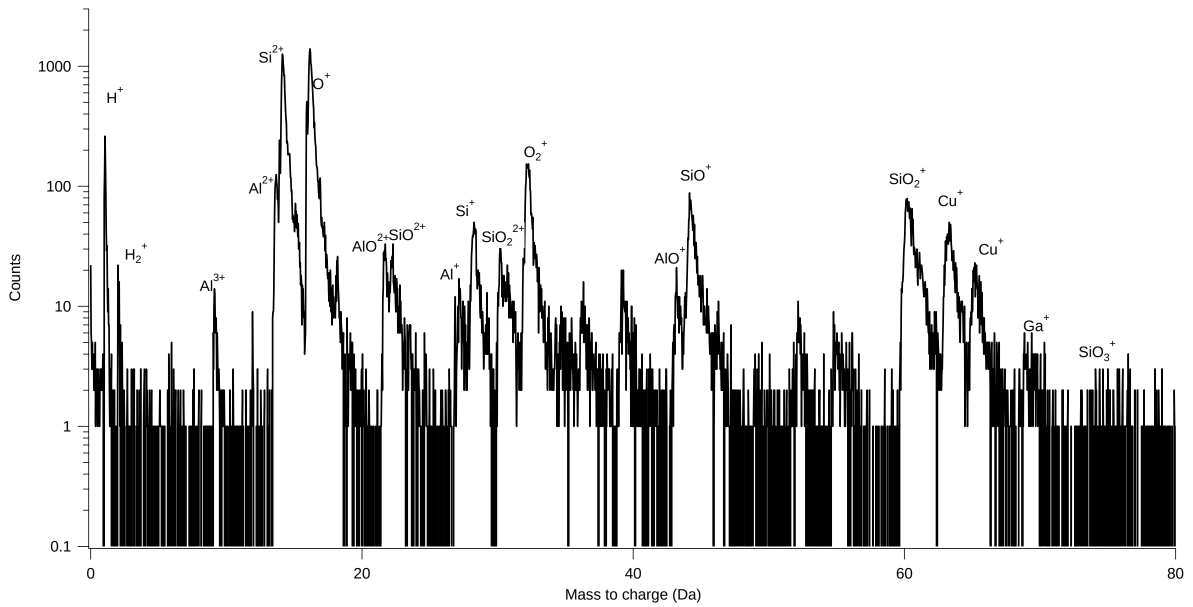

Supplementary Figure 9. TOF-MS data scale for aged Cu-ZSM-5 (sample 3) before exclusion of Ga rich region.

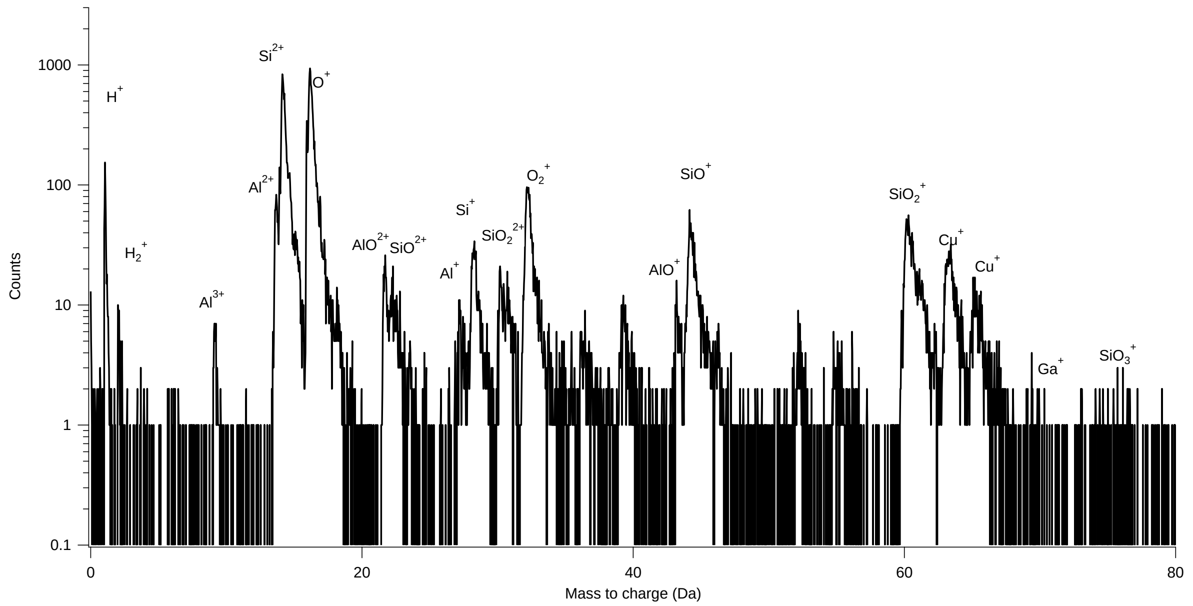

Supplementary Figure 10. TOF-MS data for aged Cu-ZSM-5 (sample 3) after exclusion of Ga rich region.

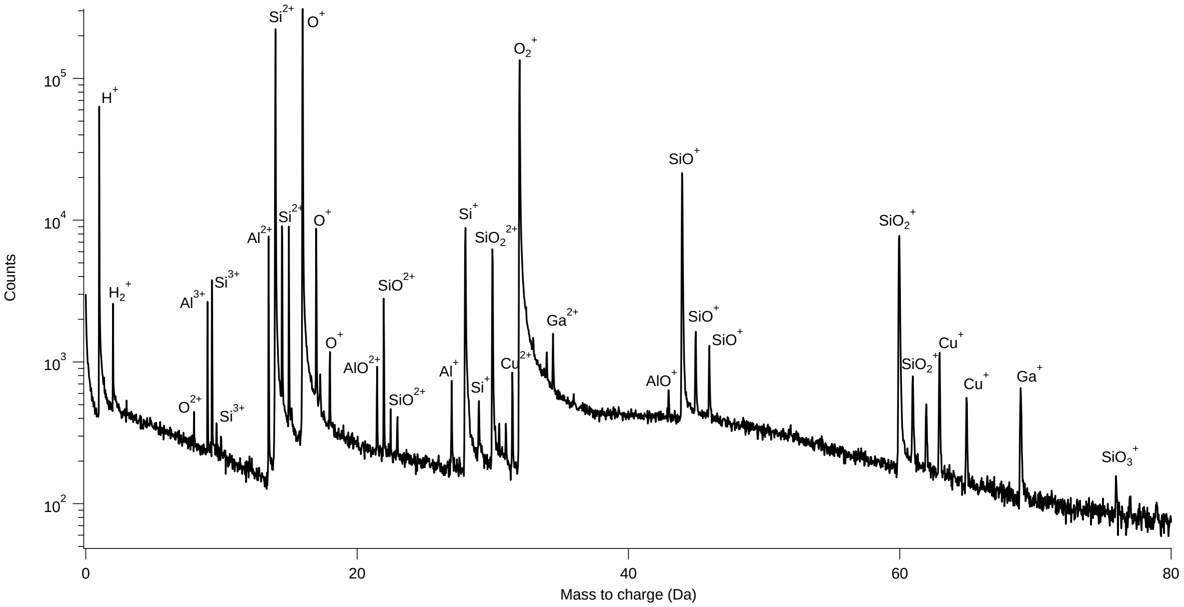

Supplementary Figure 11. TOF-MS data for fresh Cu-SSZ-13 (sample 4) before exclusion of Ga rich region.

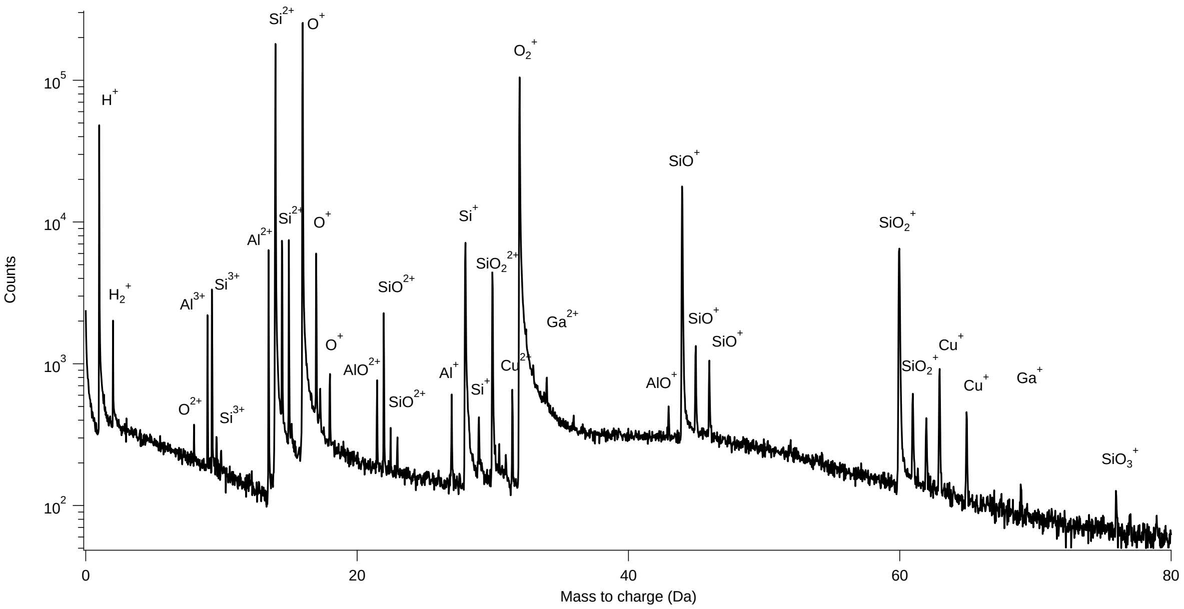

Supplementary Figure 12. TOF-MS data for fresh Cu-SSZ-13 (sample 4) after exclusion of Ga rich region.

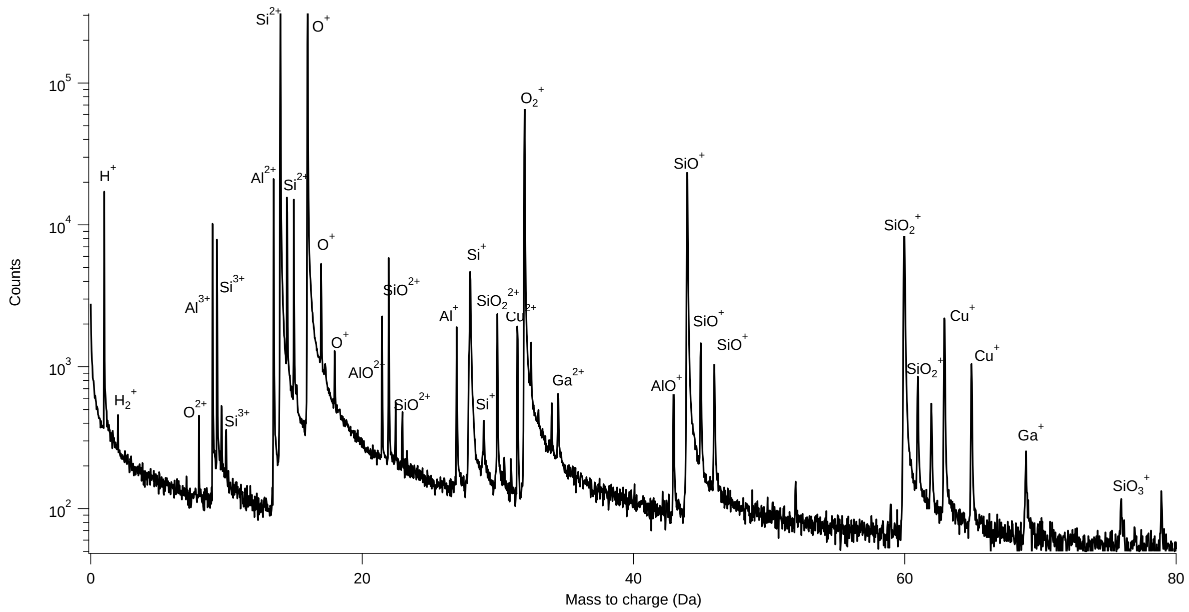

Supplementary Figure 13. **TOF-MS data for aged Cu-SSZ-13 (sample 6)** before exclusion of Ga rich region.

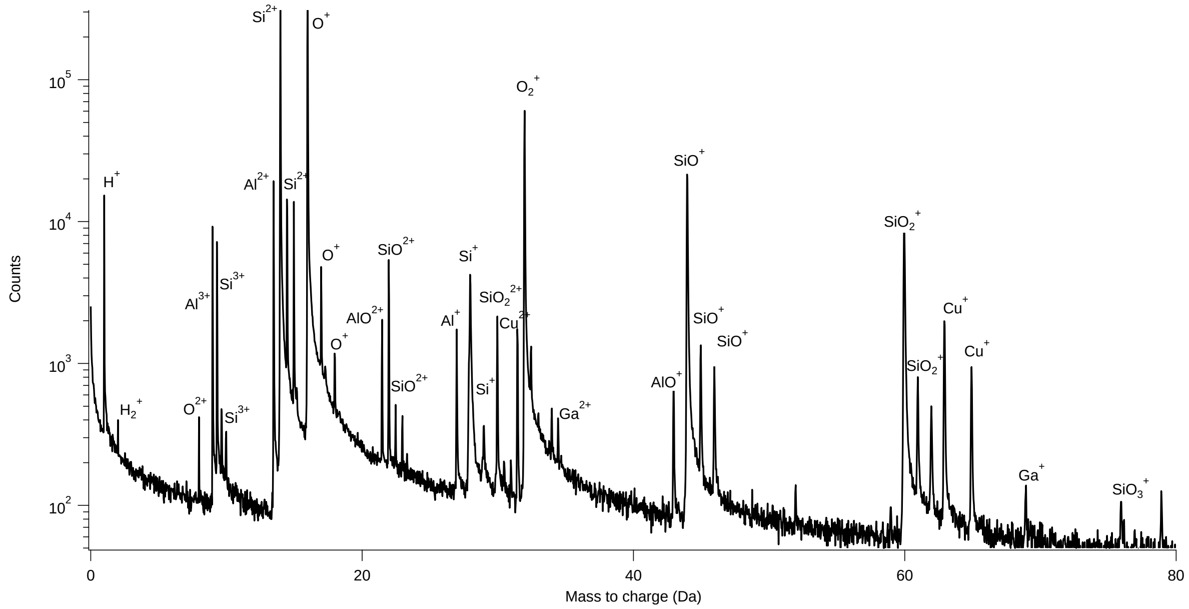

Supplementary Figure 14. TOF-MS data for aged Cu-SSZ-13 (sample 6) after exclusion of Ga rich region.

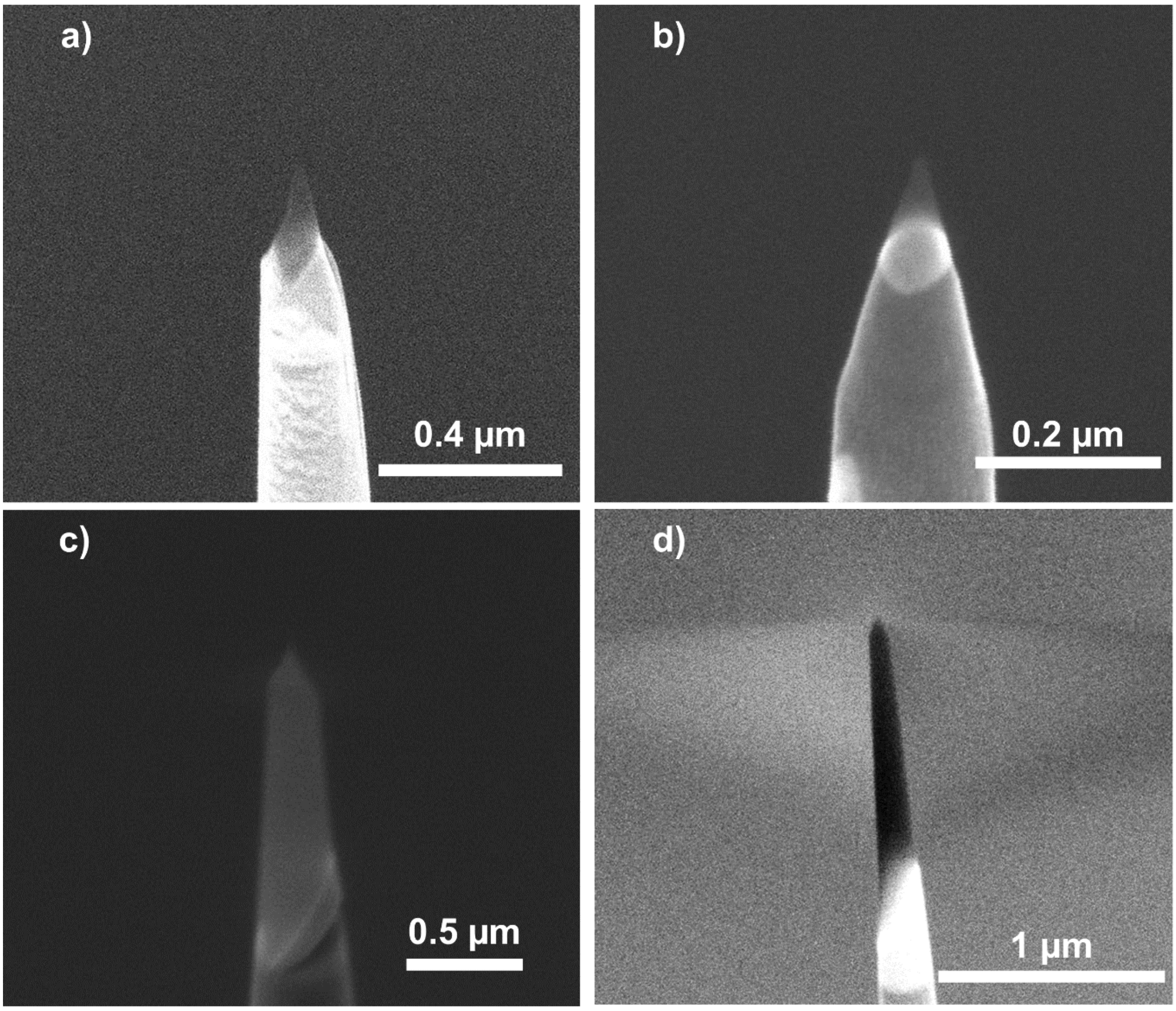

Supplementary Figure 15. Scanning electron microscopy (SEM) images of **a**, fresh Cu-ZSM-5 (sample 1), **b**, aged Cu-ZSM-5 (Sample 3), **c**, fresh Cu-SSZ-13 (sample 4) and **d**, aged Cu-SSZ-13 (sample 6) prior to the APT experiment to illustrate the differences between the tip shapes that may account for differences in the mass spectra.

Isoconcentration surface analysis

Isoconcentration surface analysis is extensively discussed in reference [9] and the references contained therein. The isoconcentration surface analysis is initiated by creating a 3D grid in concentration space. After this grid is created, 3D surfaces connecting points of equal elemental concentration can be generated, so called isoconcentration surfaces. These surfaces allow internal features to be identified, and may be defined by a single element or multiple elements. The 3D grid is created by defining a voxel size and delocalization, with the tradeoff that smaller voxels will give a higher spatial resolution but a greater amount of noise. Once the 3D grids are generated, relevant isoconcentration surfaces can be found using concentration thresholds. The importance of the isoconcentration surface analysis comes in evaluating the resulting proximity histograms, which allow the border between concentration regions to be quantitatively evaluated. The exact boundary value chosen is not important as it can change with voxel size and delocalization, what is important is that the proximity histograms allow regions that are poor or rich in specific ion(s) to be identified and separated. We generally used the default settings in IVAS (3x3x3 nm delocalization and 1 nm voxel size). However, there were instances in which the voxel size was reduced to create a higher polygon density to increase the signal to noise ratio because the edge polygons are not included in the proximity histogram analysis. The delocalization remained the same, so the shape of the interfaces was not significantly altered with the reduced voxel size.

Assessing Ga damage

It is well known in APT that Ga damage can be problematic as it may influence the position of ions, making any findings the result of needle fabrication rather than an actual material property, potentially leading to incorrect conclusions. In order to minimize this during zeolite sample preparation, we coated the samples with Pt using ebeam deposition. A 30 kV beam was used for the initial milling, leaving an ~250 nm wide needle with several hundred nm's of Pt remaining on the surface. A 2 kV final milling step was used to remove the Pt cap. The zeolite material mills much faster than the Pt cap, and therefore, the tip shape was finally a very sharp needle with a smaller shank angle. We were also careful to minimize the ebeam exposure to the material because zeolites also suffer from damage resulting from the ebeam.

Potential Ga damage has been an issue that we were also concerned with when analyzing the data, so we used a Ga isoconcentration surface to remove the Ga rich portions of the needle (normally the needle tip and surface) prior to any analyses by exporting the low gradient side of the Ga interface. The figures show the full datasets for aesthetic purposes. To demonstrate that Ga was not the cause of the major material features we observe, we show compositional histograms for all isosurfaces included in the figures of the main manuscript, but plotted in atomic % and with the Ga concentration included (Supplementary Figure 16), and the RDFs for needle 4 (Supplementary Figure 17.) as it did not have any isoconcentration surfaces. As these analyses show, there was no significant increase in Ga corresponding with Al and/or Cu agglomeration, demonstrating that these material features are not created during FIB milling.

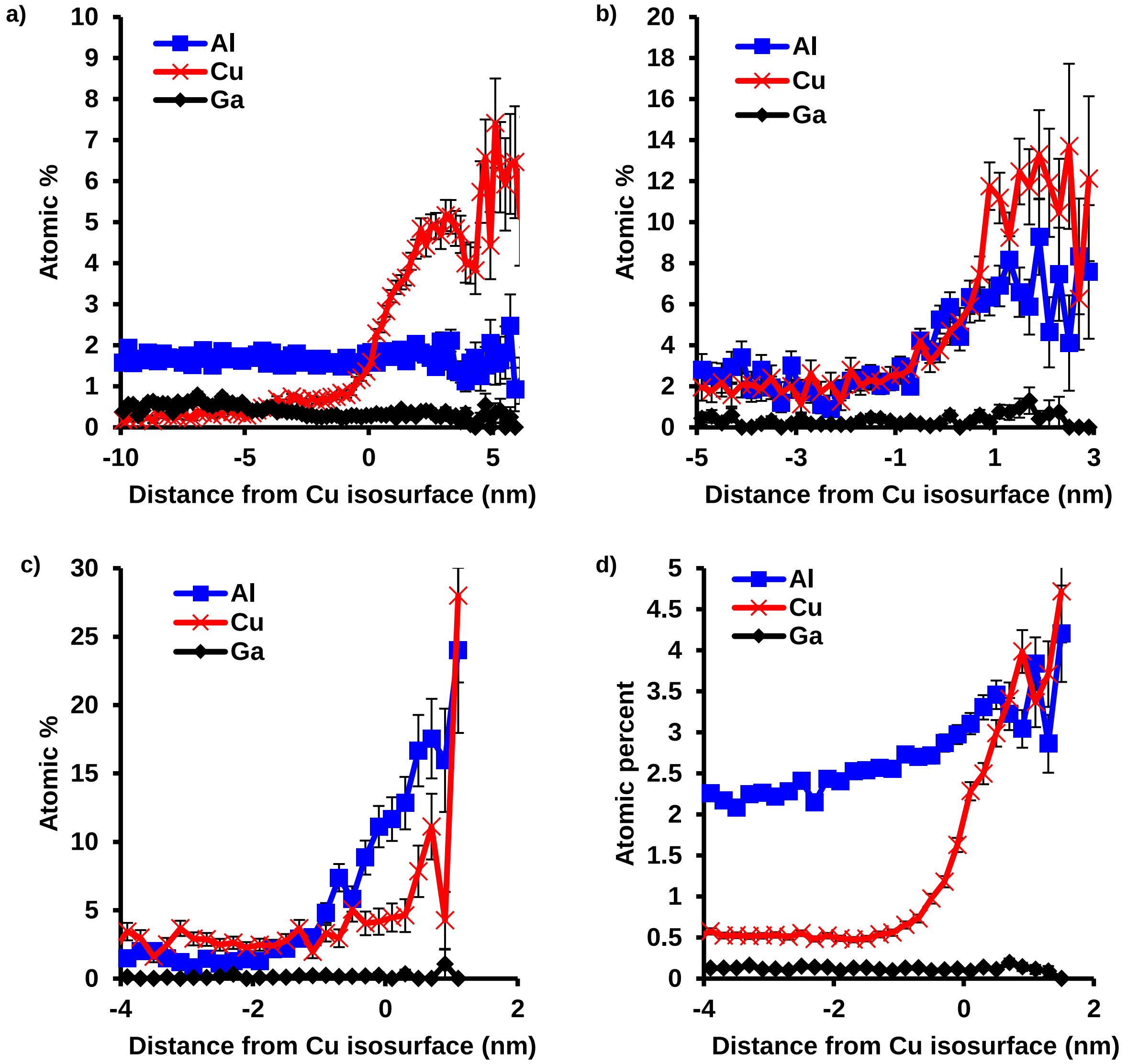

Supplementary Figure 16. Compositional histograms including Ga concentration and plotted in atomic % for all isoconcentration surfaces discussed in the manuscript. **a,** 1.8% Cu isoconcentration surface in needle 1. **b,** 5% Cu isoconcentration surface in needle 3. **c**, 8% Al isoconcentration surface in needle 3. **d**, 1.4% Cu isoconcentration surface in needle 6. All error bars were calculated from counting statistics using the method described in reference [8].

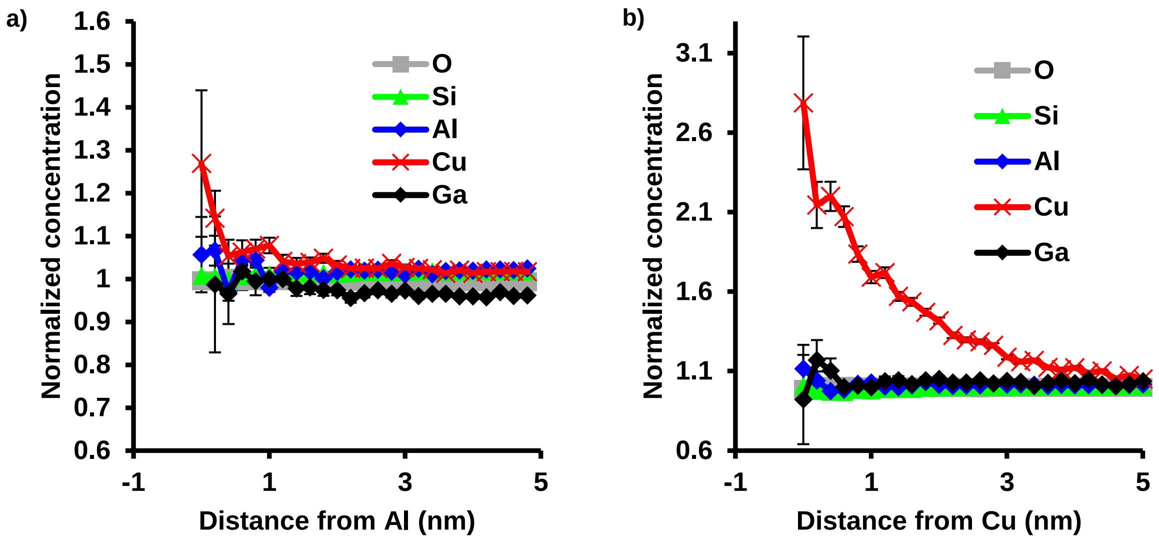

Supplementary Figure 17. Radial distribution function (RDF) for Al (a) and Cu (b) in fresh Cu-SSZ-13 (needle 4) indicating there is no affinity between Ga and Al or Cu. All error bars were calculated from counting statistics using the method described in reference [8].

Cluster analysis

The purpose of cluster analysis is to identify regions where the spacing between solute atoms in APT data is smaller than that in the bulk, effectively determining regions that are locally enriched in a specified element. For a detailed discussion of the procedure the reader is referred to reference [9]. As a summary, cluster analysis is initialized by choosing a N_{min} value, which is the minimum number of solute atoms that can form a cluster. With N_{min} set, different values of D_{max} can be evaluated. D_{max} defines the maximum distance in which another solute atom must be found to form a cluster (one additional solute atom must be found for order = 1, higher orders are also possible with more than one solute atom required to fall within D_{max}). A minimum value of D_{max} is established such that clusters containing at least N_{min} atoms are identified. There are additional parameters that are used in the analysis, and matrix atoms contained in the cluster can also be identified. The key element of cluster analysis is how the parameters are determined and significant clustering is identified. The significance test is accomplished by first taking the number of solute atoms and volume being considered, and randomizing their position using a normal distribution in space. Then, the same cluster analysis is carried out on the randomized data. Once these two data sets are established, the cluster parameters are determined using an iterative process where N_{min} is first set and then the cluster count is plotted as a function of D_{max}. If the solute data is found to be significantly separated from the randomized data (normally chosen as a point where clusters exist in the collected data and zero, or a very minimal amount, such that >95% of the clusters would be non-random, exist in the randomized data), then D_{max} can be fixed. A fixed D_{max} can then be used to plot cluster count versus cluster size to set an optimal N_{min}. By iterating through these parameters, the optimal cluster D_{max} and N_{min} can be determined, where significant clusters can be found relative to the randomized data. This iterative process as applied through the cluster count distribution (CCD) and cluster size distribution (CSD) as applied to the data sets in this manuscript are shown with the analyses for each individual parameter along with the other parameters determined for each specific cluster analysis.

A simple method that is useful in identifying if clusters are present and a cluster analysis should be initiated, is by directly comparing the nearest neighbor distributions (NNDs) of the solute and randomized data[12]. In doing this, the number of counts is plotted as a function of the atom pair distance. For the randomized data this will, by definition, give a normal distribution. However, if clustering is present, then the solute NNDs will be shifted to a smaller atom pair distance. The NND for the solute data is then described best by at least two Gaussians, one centered at a smaller atom pair distance, representing the clusters, and a second centered at an atom pair distance greater than the normal distribution of the randomized data, representing the matrix ion spacing. This makes plotting the NNDs an invaluable tool in cluster analysis.

In this work, when performing the Al cluster analysis, we used all Al related peaks (Al and AlO). For Cu, there were only metallic Cu peaks and all of those were used in the Cu cluster analysis.

Radial distribution function

The radial distribution function (RDF) is a powerful tool to examine affinity between small numbers of atoms and test homogeneity, and it was applied using CAMECA's IVAS software. The method is extensively discussed in references [13] and [14]. The RDF is conducted by normalizing the local concentration of a selected ion by the bulk concentration, and this is done radially outward from the center of the ion(s) of interest. Error analyses were conducted using counting statistics; because few ions will be counted near each ion of interest, the error bars are high for the first few data points. As the RDF is shown as a bulk normalized concentration, values between pairs of atoms will not be equal. For example, Cu-Al is not equal to Al-Cu. The number of counts is equal for both pairs, but not the bulk normalized concentrations since they are defined over different volumes. This is illustrated in 2D below in Supplementary Figure 18. In our analyses, all molecular ions were decomposed prior to performing the RDF.

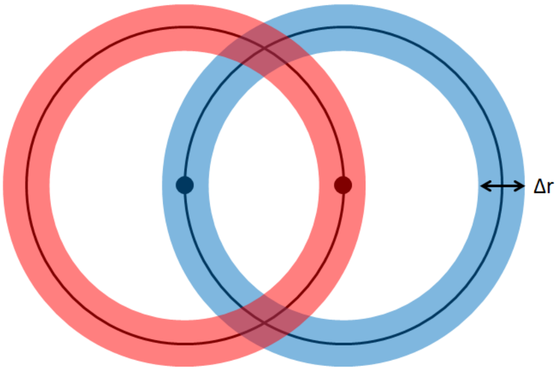

Supplementary Figure 18. **2D schematic of the radial distribution function (RDF)** for analyzing the spatial distribution of different elements in the atom probe tomography (APT) data.

APT data analysis workflow

The processing and analysis of the data obtained in an APT experiment is far from trivial and is the subject of active research and debate[9,15–17]. Below is a brief outline of the normal workflow used in the processing of APT data as performed for the experiments described herein. This is not meant to be an exhaustive or definitive guide, but rather a brief overview in order to give the reader an idea of the process used, and the reader is referred to our previous manuscript for further details[10].

1. Data collection with an APT instrument.
2. Data processing and reconstruction: When processing zeolite samples it is especially important that all peaks are ranged correctly. The 3D distribution of all ions can then be generated.
3. Identification of isoconcentration surfaces: Isoconcentration surface analysis is used to separate large compositional inhomogenities. The isoconcentration surfaces are quantified using proximity histograms.
4. Cluster identification: Within subvolumes (if any exist, isolated using isoconcentration surface analysis), both the nearest neighbor distributions (NNDs) and frequency distribution analysis (FDA) are used to examine compositional heterogeneities that are not as obvious as isoconcentration surfaces. The identification of clusters is an iterative process between comparing the cluster count distribution (CCD) and cluster size distribution (CSD) so that statistically relevant clusters can be identified.
5. The radial distribution function can be used to examine compositional heterogeneities that exist that may not have been identified using isoconcentration surface analysis or cluster analysis.

APT sample reconstructions

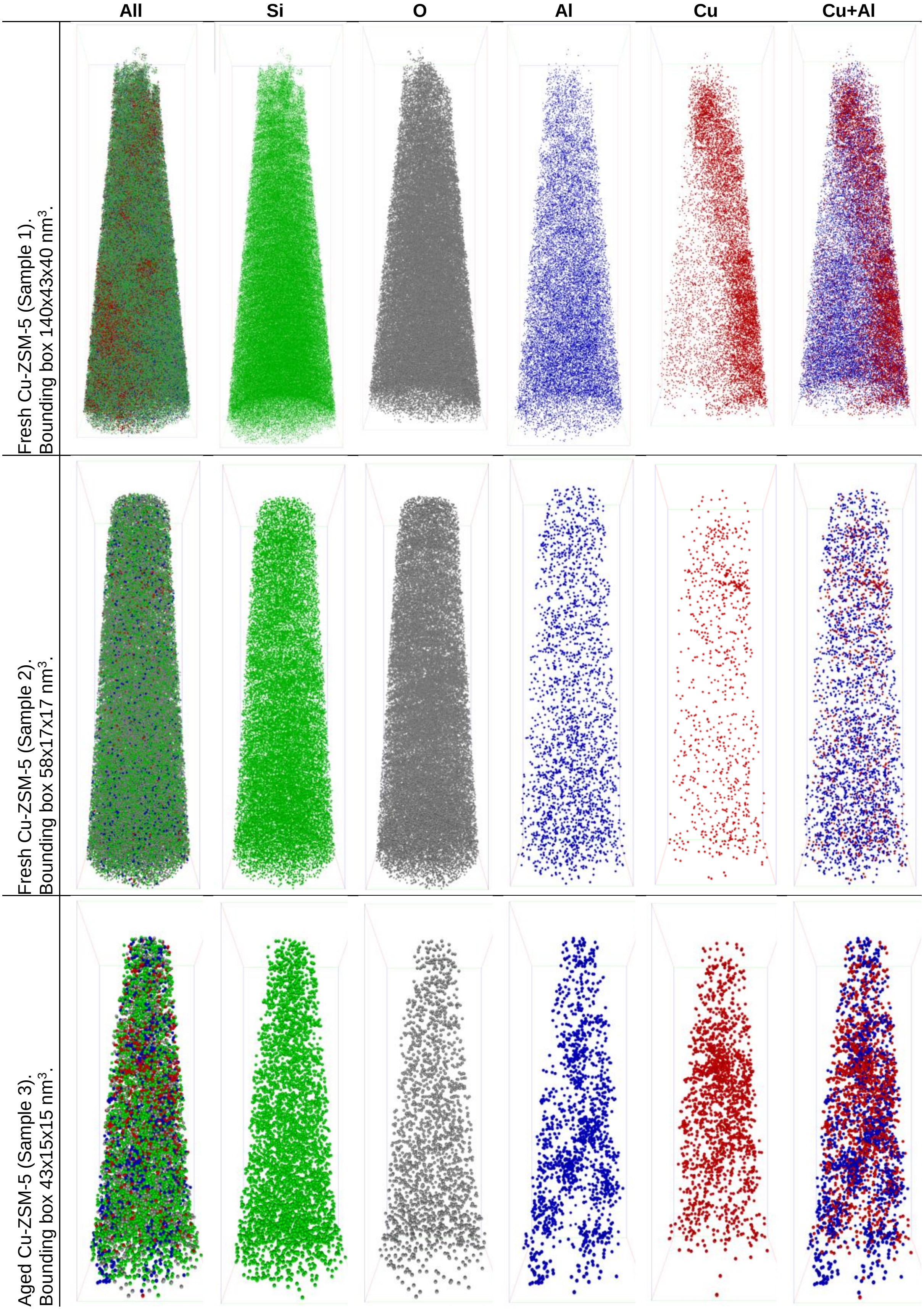

Supplementary Figure 19. Reconstructions of fresh and aged Cu-ZSM-5 needles.

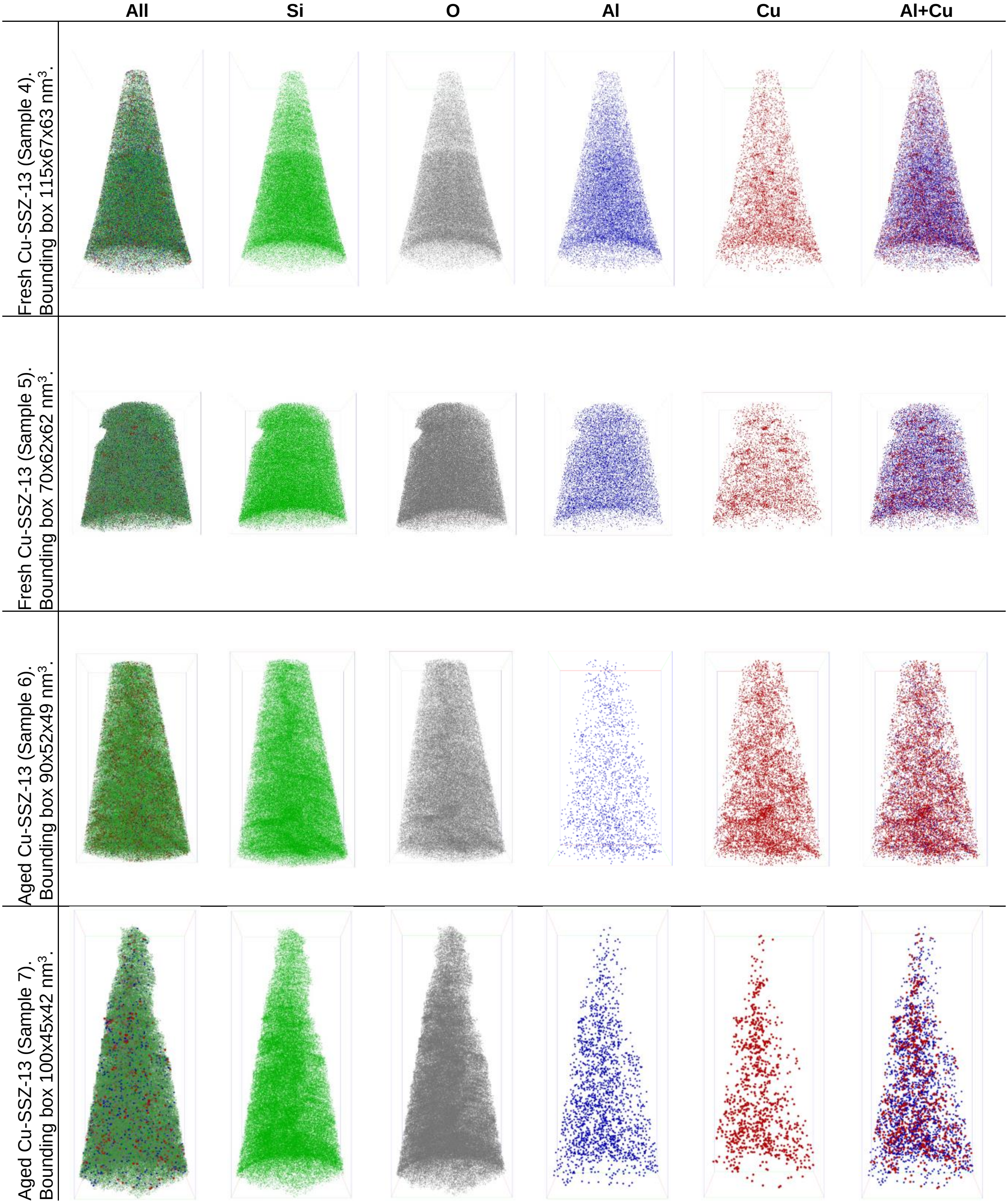

Supplementary Figure 20. Reconstructions of fresh and aged Cu-SSZ-13 needles.

Fresh Cu-ZSM-5 (Sample 1). Bounding box 140x43x40 nm^3.	Aged Cu-ZSM-5 (Sample 3). Bounding box 43x15x15 nm^3.	Fresh Cu-SSZ-13 (Sample 4) Bounding box 115x67x63 nm^3.	Aged Cu-SSZ-13 (Sample 6) Bounding box 90x52x49 nm^3.

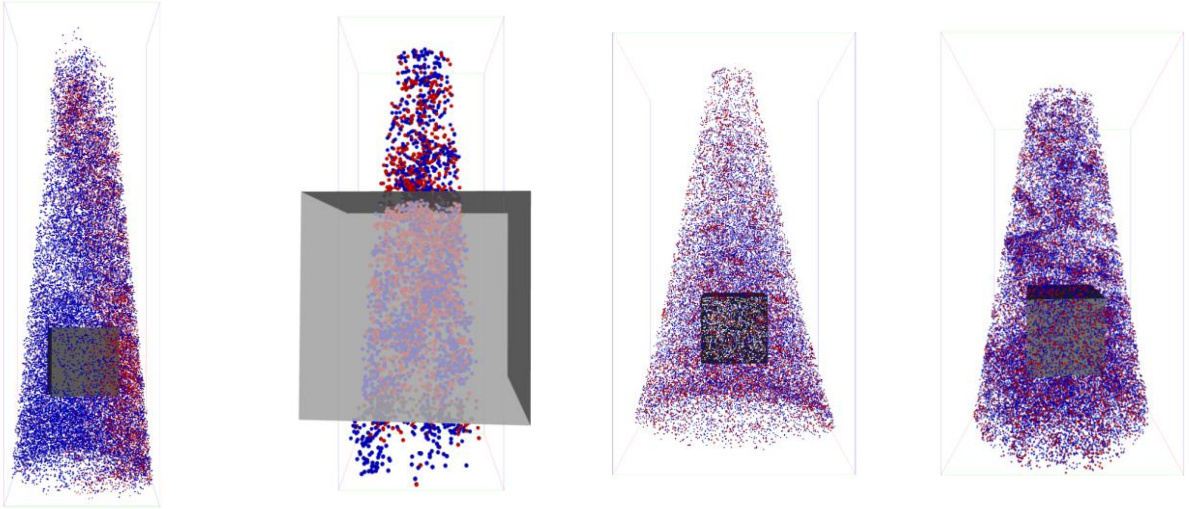

Supplementary Figure 21. Locations of 20x20x20 nm regions of interest shown in Fig. 4.

Supplementary Table 1. Compositions of needles determined by APT before and after the Ga rich region was extracted. A background correction was performed for all compositions. All compositions are in atomic percent.

**Complete data set**							
	Fresh Cu-ZSM-5	Fresh Cu-ZSM-5	Aged Cu-ZSM-5	Fresh Cu-SSZ-13	Fresh Cu-SSZ-13	Aged Cu-SSZ-13	Aged Cu-SSZ-13
Sample #	**1**	**2**	**3**	**4**	**5**	**6**	**7**
Al	1.8	2.5	3.5	0.8	0.9	2.4	2.0
O	61.8	55.2	58.2	74.0	72.2	62.2	64.5
Si	35.7	41.3	35.6	23.9	26.5	34.5	32.6
Cu	0.4	0.7	2.3	0.3	0.2	0.7	0.5
Ga	0.3	0.4	0.3	0.2	0.1	0.1	0.5
Si/Al	19.6	16.7	10.3	30.6	28.8	14.5	16.6
Cu/Al	0.2	0.3	0.7	0.4	0.3	0.3	0.2
**After removal of Ga rich region**							
	Fresh Cu-ZSM-5	Fresh Cu-ZSM-5	Aged Cu-ZSM-5	Fresh Cu-SSZ-13	Fresh Cu-SSZ-13	Aged Cu-SSZ-13	Aged Cu-SSZ-13
Sample #	**1**	**2**	**3**	**4**	**5**	**6**	**7**
Al	1.8	-	3.3	0.8	0.9	2.4	1.9
O	61.7	-	57.9	73.6	72.8	62.2	66.4
Si	35.8	-	36.0	25.3	26.1	34.6	31.2
Cu	0.6	-	2.6	0.3	0.3	0.7	0.5
Ga	0.1	-	0.1	0.0	0.0	0.0	0.1
Si/Al	20.1	-	11.0	31.3	28.7	14.5	16.8
Cu/Al	0.3	-	0.8	0.4	0.3	0.3	0.2

Individual analyses

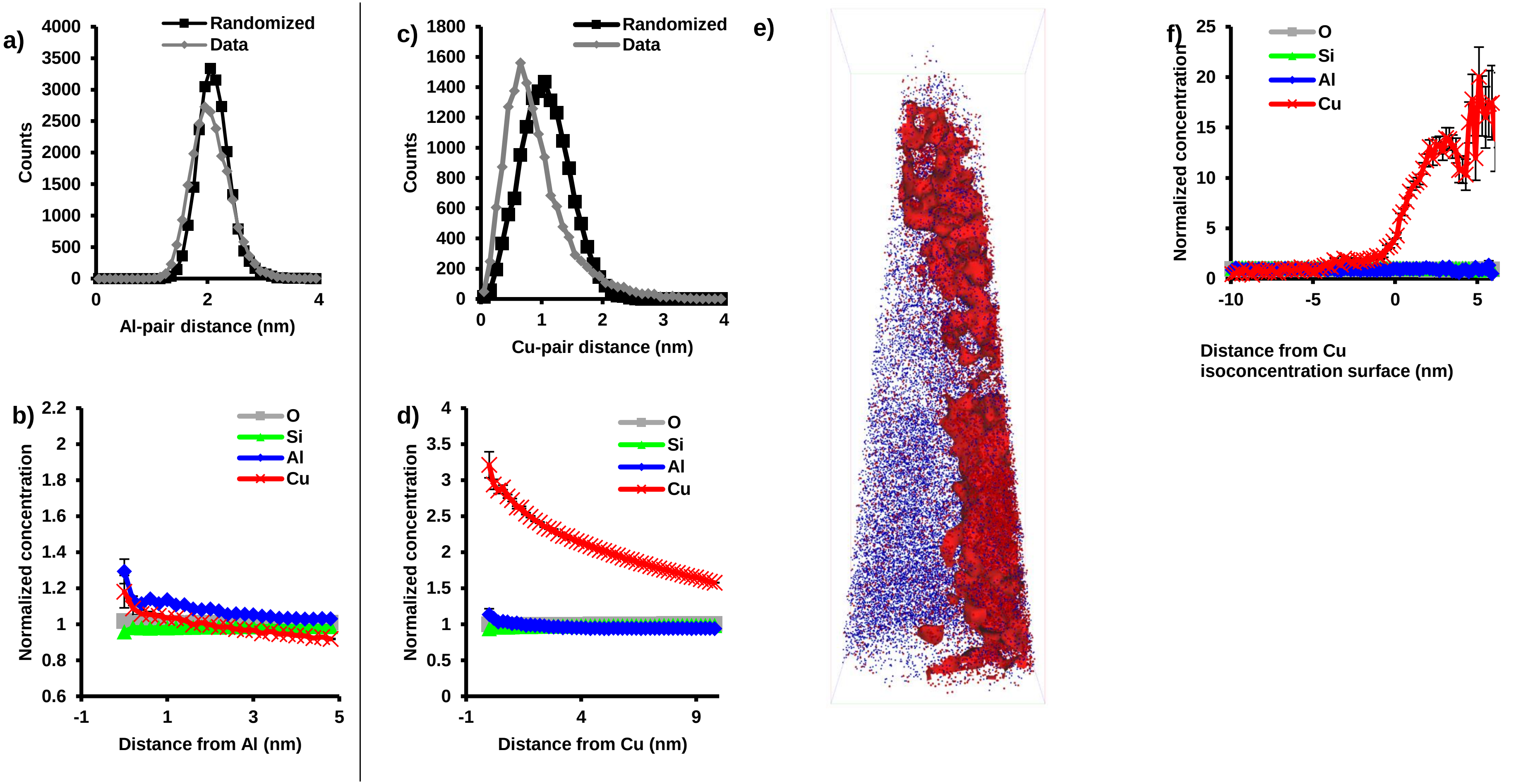

Supplementary Figure 22. Analysis of fresh Cu-ZSM-5, sample 1: a, Al nearest neighbor distribution (NND, order = 10). **b,** Al radial distribution function (RDF). **c,** Cu NND. **d,** Cu RDF. **e,** 1.8% Cu isoconcentration surface shown with all Al (blue) and Cu (red) ions. Bounding box dimensions of 140x43x40 nm^3. This image is also included as Supplementary Movie 1. **f,** Bulk normalized compositional histograms across the 1.8% Cu isoconcentration surface. All error bars were calculated from counting statistics using the method described in reference [8].

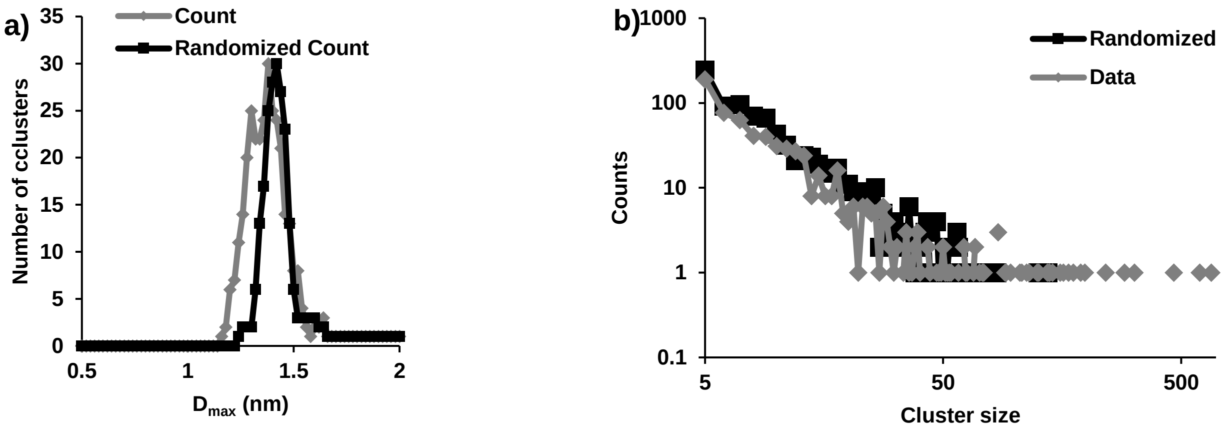

Supplementary Figure 23. Al distribution analysis of fresh Cu-ZSM-5, sample 1: a, Cluster count distribution. **b,** Cluster size distribution, note log-log axes are used to show there are large clusters.

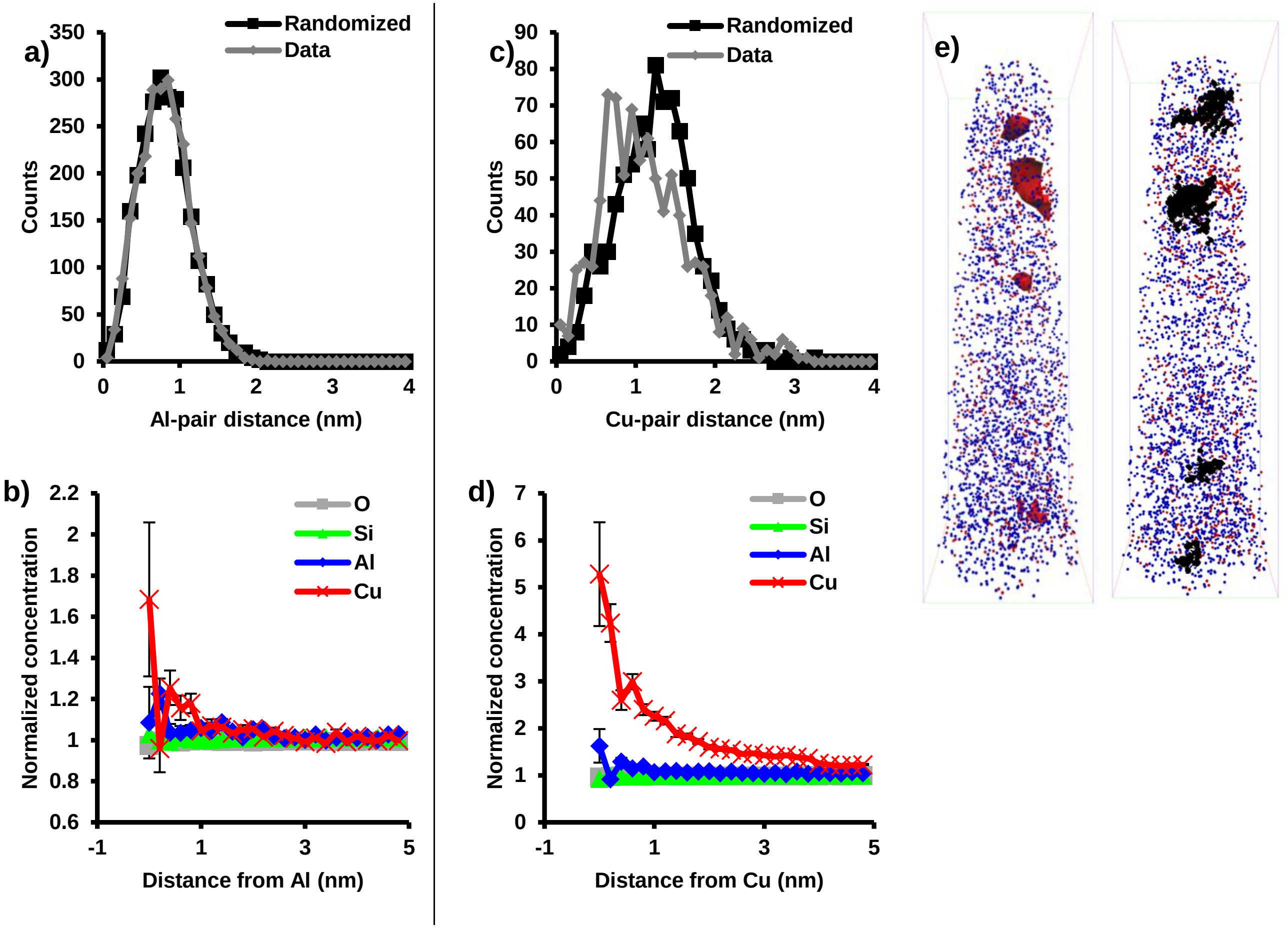

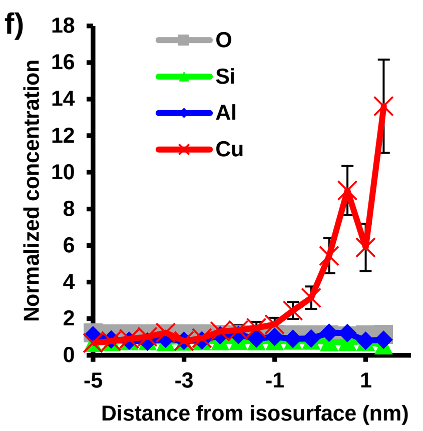

g)

Ion	At. % Bulk	At. % Cluster
O	54.0%	55.5%
Si	42.7%	30.9%
Al	2.5%	9.9%
Cu	0.4%	2.6%
Si/Al	17.1	3.1
Cu/Al	0.2	0.3

Supplementary Figure 24. Analysis of fresh Cu-ZSM-5, sample 2: a, Al nearest neighbor distribution (NND). **b,** Al radial distribution function (RDF). **c,** Cu NND. **d,** Cu RDF. **e,** (left) 2.7% Cu isoconcentration surface shown with all Al (blue) and Cu (red) ions. (right) Map of Cu clusters in black overlaid over all Al and Cu ions. Bounding box dimensions of 58x17x17 nm^3. These images are also included as Supplementary Movie 2. **f,** Bulk normalized compositional histograms across the 2.7% Cu isoconcentration surface. **g,** Bulk versus cluster composition. All error bars were calculated from counting statistics using the method described in reference [8].

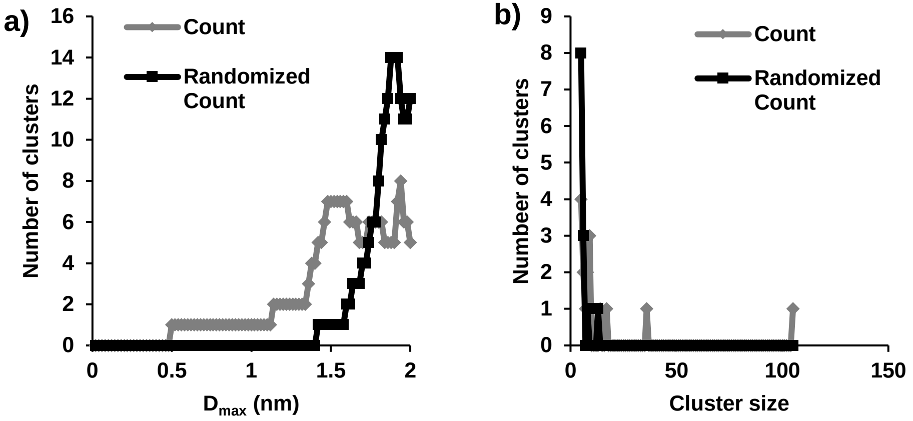

c)

ROI	Top-Level ROI
Ion(s)	Cu
D_{max} (nm)	1.4
Order (ions)	4
N_{min} (ions)	14
L (nm)	1.4
$D_{erosion}$ (nm)	1.12
Cluster Count	4

Supplementary Figure 25. Cu cluster analysis of fresh Cu-ZSM-5, sample 2: a, Cluster count distribution. **b,** Cluster size distribution. **c,** Table of analysis parameters used in the cluster analysis.

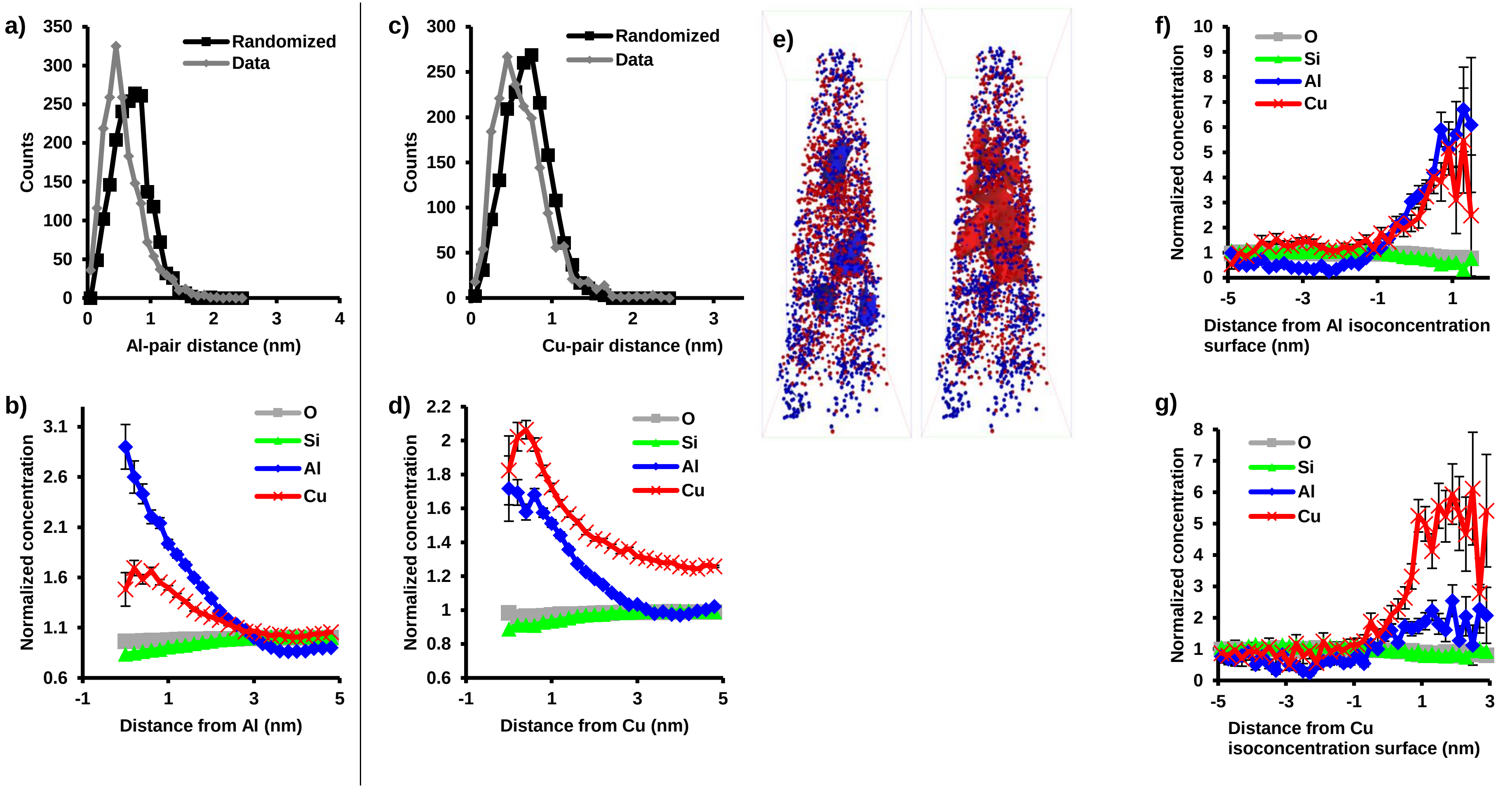

Supplementary Figure 26. Analysis of aged Cu-ZSM-5, sample 3: a, Al nearest neighbor distribution (NND). **b**, Al radial distribution function (RDF). **c**, Cu NND. **d**, Cu RDF. **e**, (left) 8% Al isoconcentration surface shown with all Al (blue) and Cu (red) ions. (right) 5% Cu isoconcentration surface isoconcentration surface shown with all Al and Cu ions. Bounding box dimensions of 43x15x15 nm^3. These images are also included as Supplementary Movie 3. **f**, Bulk normalized compositional histograms across the 8% Al isoconcentration surface. **g**, Bulk normalized compositional histograms across the 5% Cu isoconcentration surface. The percentage of Cu and Al atoms within the region with Cu/Al = 2 stoichiometry (in the 8% Al isoconcentration surface) were considered to give an estimate of the portion of Cu and Al atoms forming the phase that resembles $CuAl_2O_4$.This region was determined to contain ~20 % of all Cu ions and ~35% of all Al ions present in the needle. All error bars were calculated from counting statistics using the method described in reference [8].

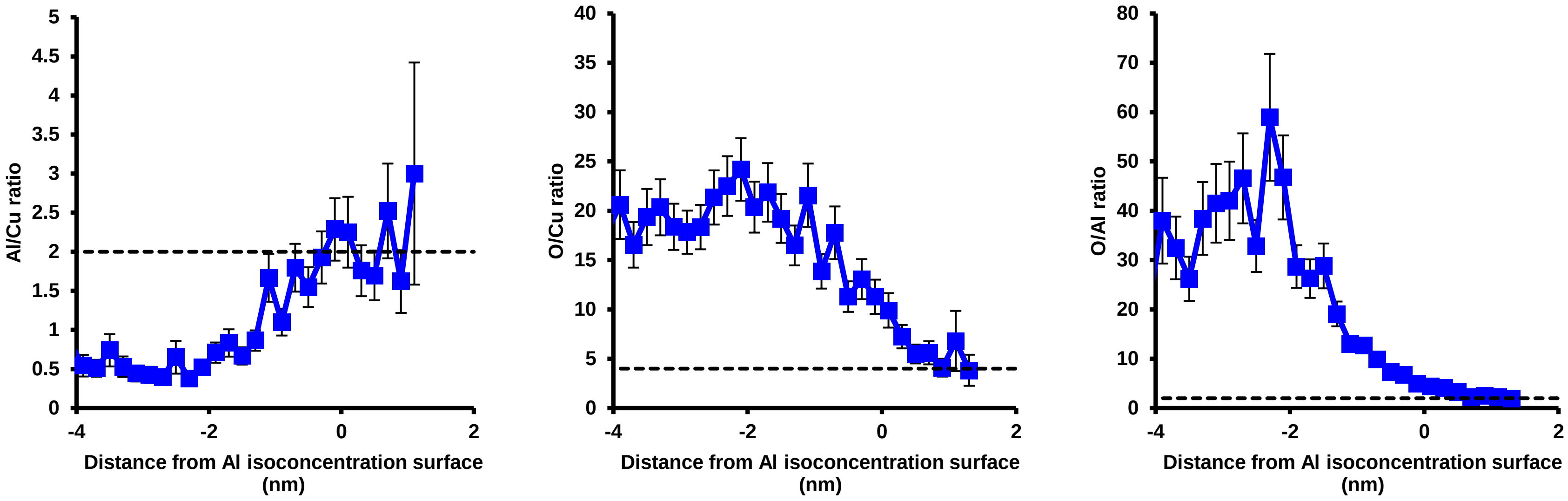

Supplementary Figure 27. Al/Cu, O/Cu and O/Al ratios across the 8% Al isoconcentration surface in aged Cu-ZSM-5 (Sample 3). Values agree with the stoichiometry of Cu aluminate spinel, $CuAl_2O_4$, which is indicated on each graph by the dotted line. All error bars were calculated from counting statistics using the method described in reference [8].

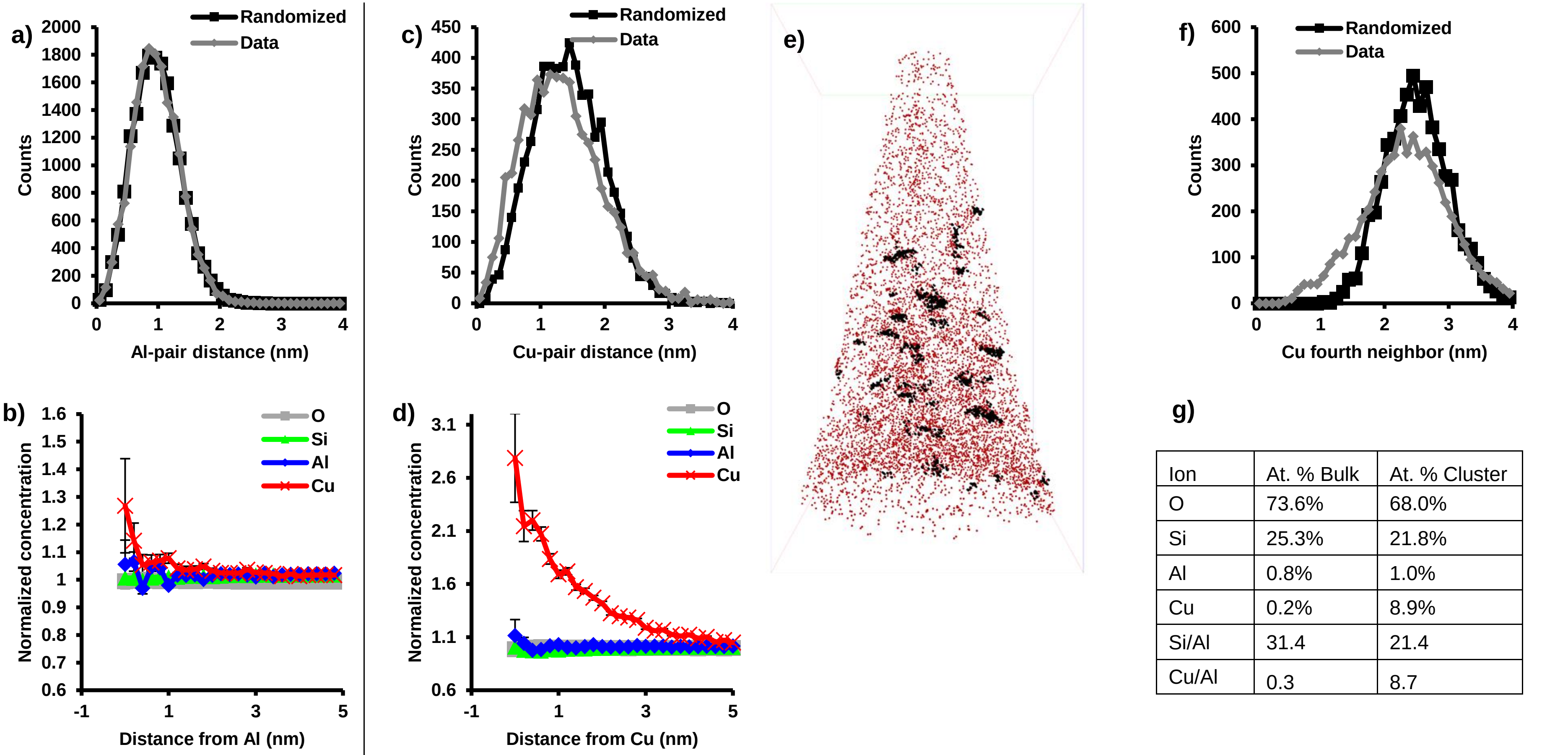

Ion	At. % Bulk	At. % Cluster
O	73.6%	68.0%
Si	25.3%	21.8%
Al	0.8%	1.0%
Cu	0.2%	8.9%
Si/Al	31.4	21.4
Cu/Al	0.3	8.7

Supplementary Figure 28. Analysis of fresh Cu-SSZ-13, Sample 4: a, Al nearest neighbor distribution (NND). **b,** Al radial distribution function (RDF). **c,** Cu NND. **d,** Cu RDF. **e,** Map of Cu clusters identified in Fresh Cu-SSZ-13 (order = 4 analysis). Cu atoms in clusters are shown in black and overlaid over all Cu ions. Bounding box dimensions of 115x67x63 nm^3. These images are also included as Supplementary Movies 4 and 5. **f,** Cu NND for the fourth nearest Cu neighbor. **g,** Bulk versus cluster composition. All error bars were calculated from counting statistics using the method described in reference [8].

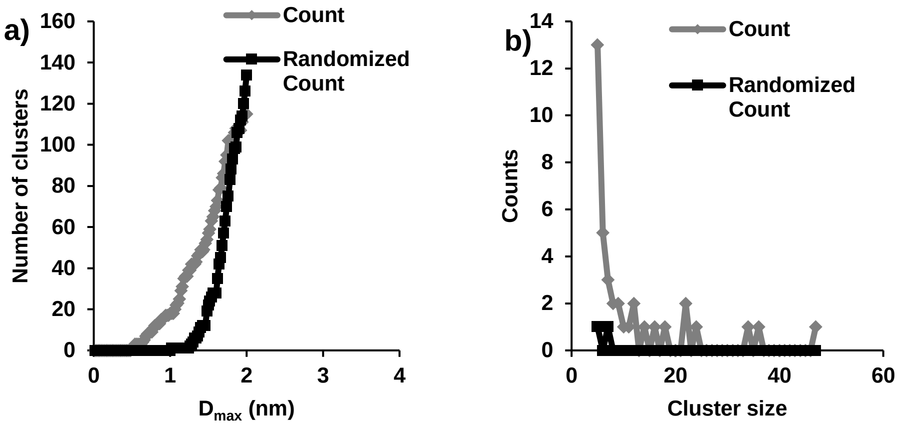

c)

ROI	Top-Level ROI
Ion(s)	Cu
D_{max} (nm)	1.3
Order (ions)	4
N_{min} (ions)	6
L (nm)	1.3
$D_{erosion}$ (nm)	1.04
Cluster Count	42

Supplementary Figure 29. Cu cluster analysis of fresh Cu-SSZ-13, sample 4: a, Cluster count distribution. **b,** Cluster size distribution. **c,** Table of analysis parameters used in the cluster analysis.

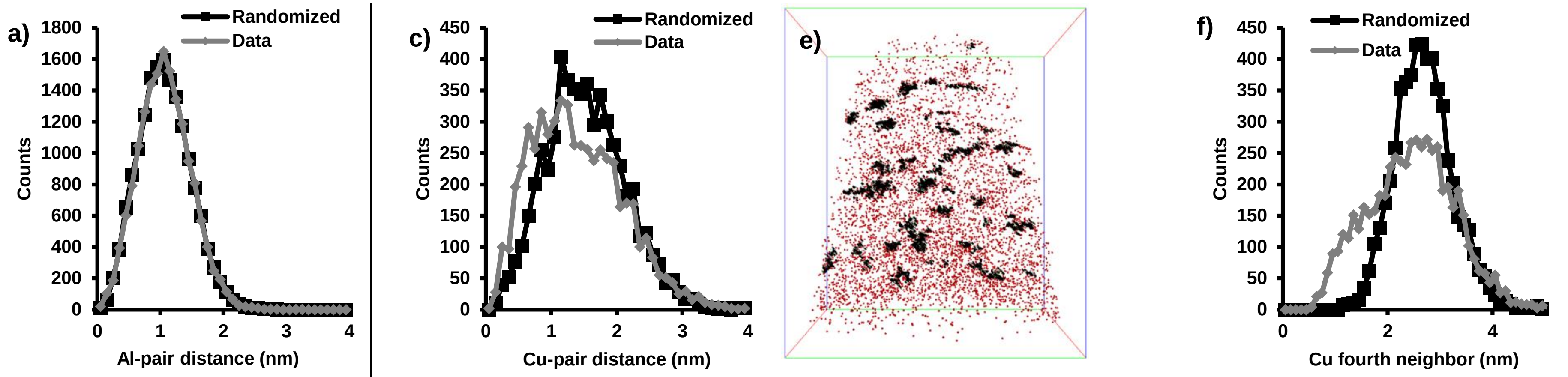

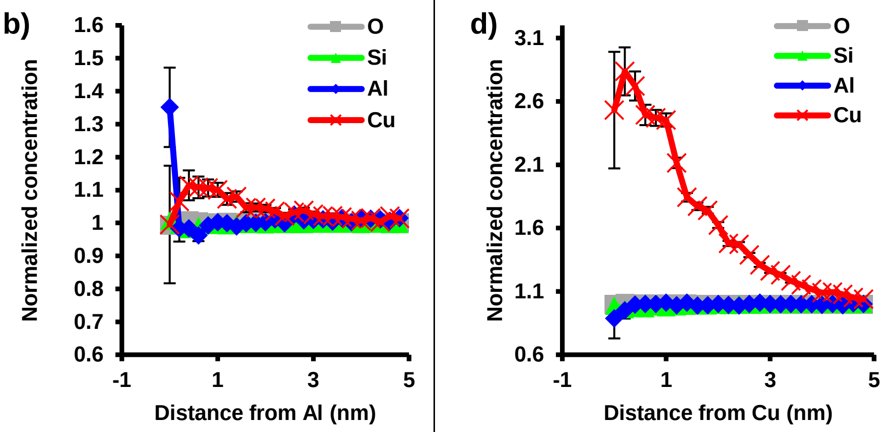

g)

Ion	At. % Bulk	At. % Cluster
O	72.8	69.6
Si	26.1	20.8
Al	0.9	8.7
Cu	0.2	1.0
Si/Al	28.7	21.6
Cu/Al	0.2	9.0

Supplementary Figure 30. Analysis of fresh Cu-SSZ-13, Sample 5: a, Al nearest neighbor distribution (NND). **b,** Al radial distribution function (RDF). **c,** Cu NND. **d,** Cu RDF. **e,** Map of Cu clusters identified in Fresh Cu-SSZ-13 (order = 4 analysis). Cu atoms in clusters are shown in black and overlaid over all Cu ions Bounding box dimensions of 70x62x62 nm^3. These images are also included as Supplementary Movies 6 and 7. **f,** Cu NND for the fourth nearest Cu neighbor. **g,** Bulk versus cluster composition. All error bars were calculated from counting statistics using the method described in reference [8].

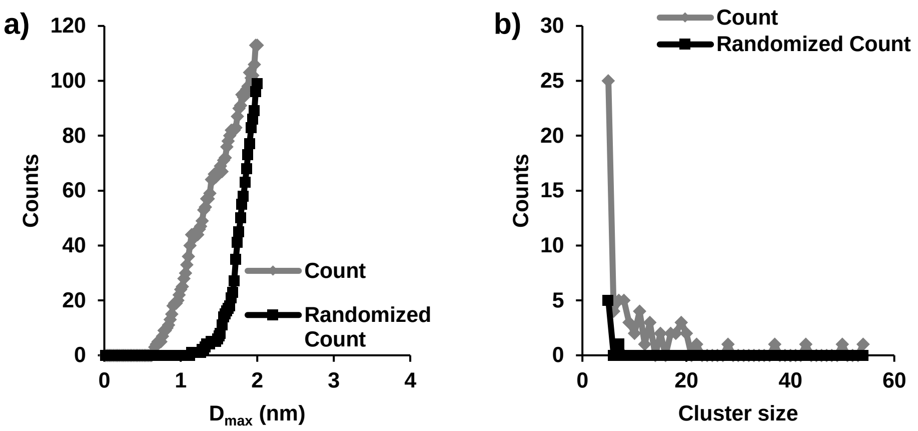

c)

ROI	Top-Level ROI
Ion(s)	Cu
D_{max} (nm)	1.38
Order (ions)	4
N_{min} (ions)	6
L (nm)	1.38
$D_{erosion}$ (nm)	1.1
Cluster Count	59

Supplementary Figure 31. Cu cluster analysis of fresh Cu-SSZ-13, sample 5: a, Cluster count distribution. **b,** Cluster size distribution. **c,** Table of analysis parameters used in the cluster analysis.

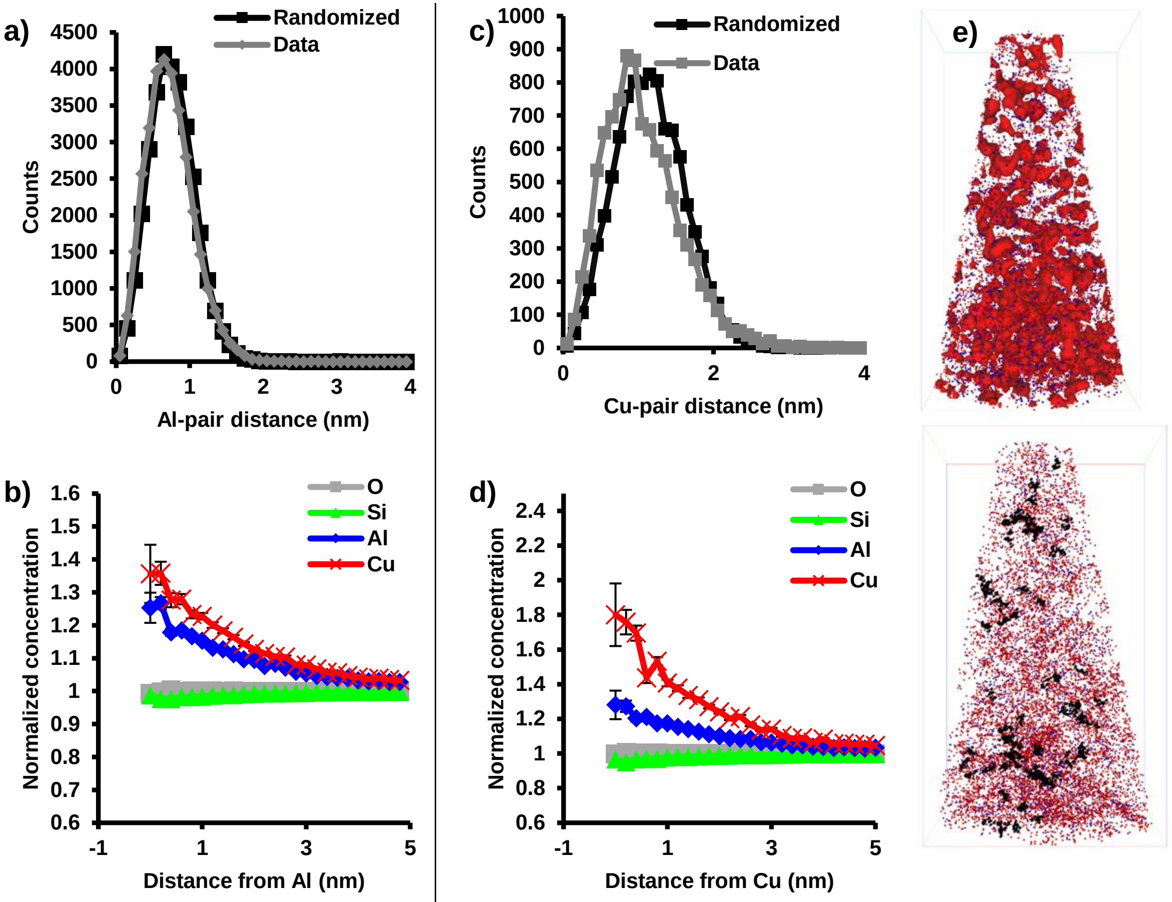

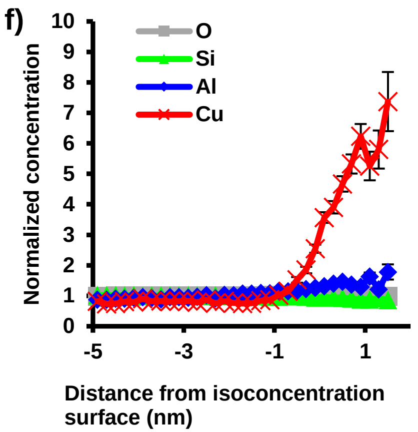

g)

Ion	At. % Bulk	At. % Cluster
O	62.5	61.2
Si	34.6	30.7
Al	2.3	4.7
Cu	0.5	3.3
Si/Al	14.9	9.2
Cu/Al	0.2	1.4

Supplementary Figure 32. Analysis of aged Cu-SSZ-13, sample 6: a, Al nearest neighbor distribution (NND). **b,** Al radial distribution function (RDF). **c,** Cu NND. **d,** Cu RDF. **e,** (top) 1.4% Cu isoconcentration surfac**e,** shown with all Al ions (blue) and Cu ions (red). (bottom) Map of Cu clusters (order = 4 analysis). Bounding box dimensions of 90x52x49 nm^3. These images are also included as Supplementary Movies 8 and 9. **f,** Compositional histograms across the 1.4% Cu isoconcentration surface g, Bulk versus cluster composition. All error bars were calculated from counting statistics using the method described in reference [8].

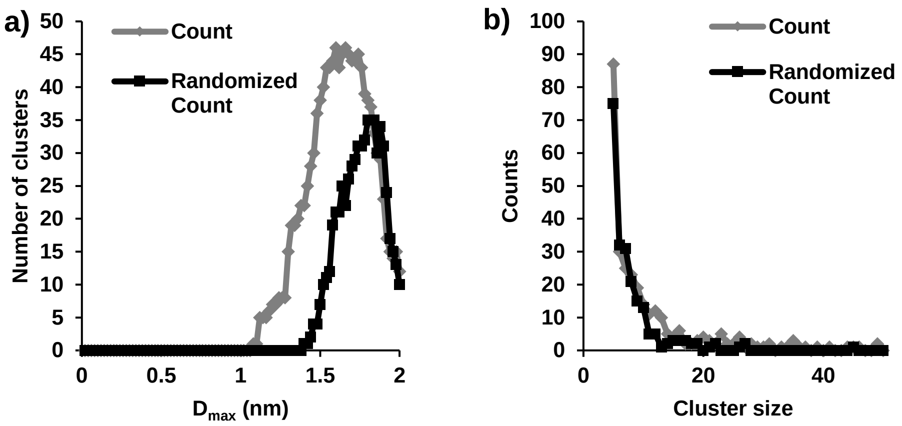

c)

ROI	Top-Level ROI
Ion(s)	Cu
D_{max} (nm)	1.48
Order (ions)	4
N_{min} (ions)	28
L (nm)	1.48
$D_{erosion}$ (nm)	1.18
Cluster Count	36

Supplementary Figure 33. Cu cluster analysis of aged Cu-SSZ-13, sample 6: **a,** Cluster count distribution. **b,** Cluster size distribution. **c,** Table of analysis parameters used in the cluster analysis.

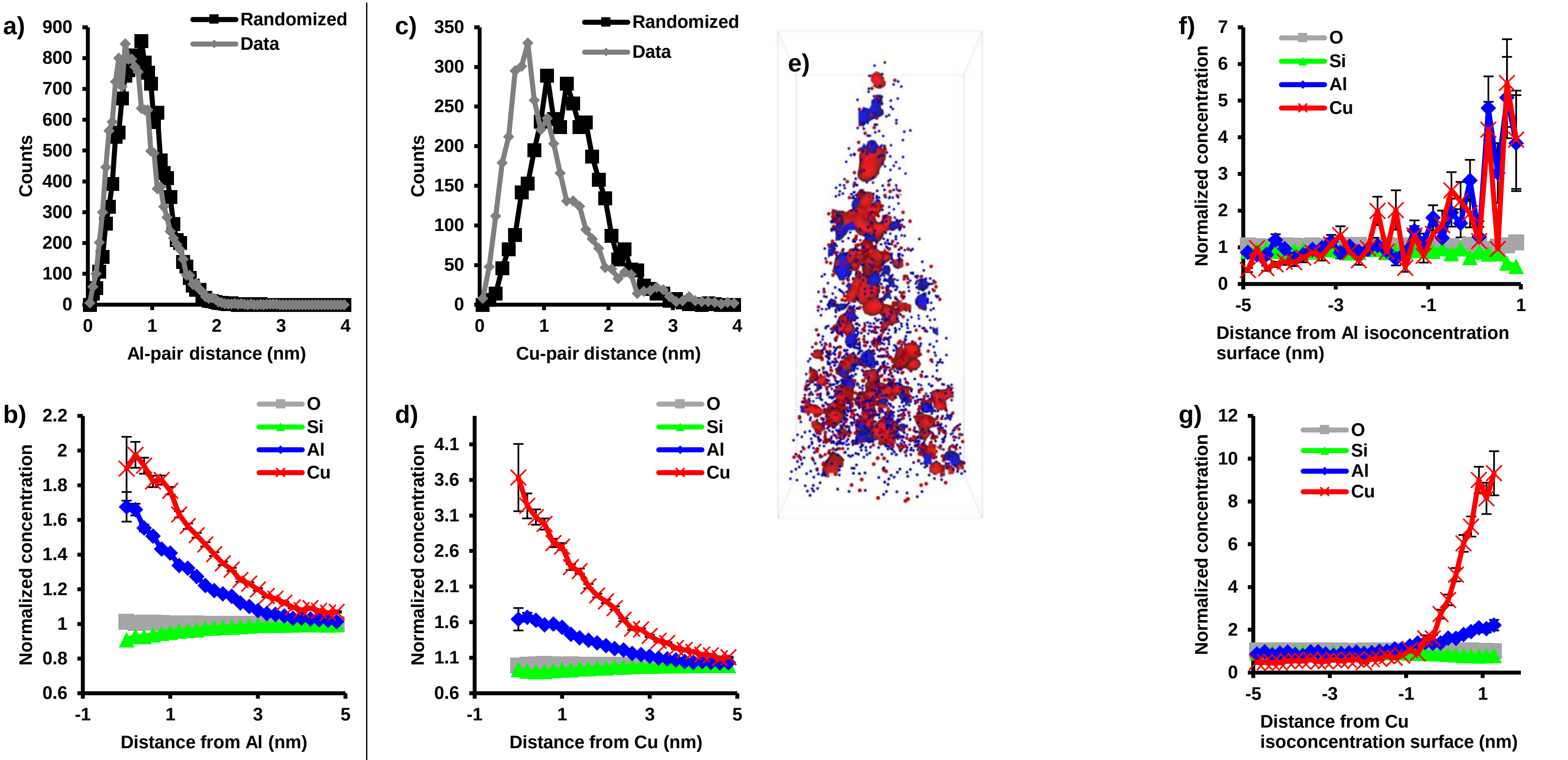

Supplementary Figure 34. Analysis of aged Cu-SSZ-13, sample 7: a, Al nearest neighbor distribution (NND). **b,** Al radial distribution function (RDF). **c,** Cu NND. **d,** Cu RDF. **e,** 1.3% Cu isoconcentration surface and 4% Al isoconcentration surface, shown with all Al ions (blue) and Cu ions (red). Bounding box dimensions of 100x45x42 nm^3. These images are also included as Supplementary Movie 10. **f,** Compositional histograms across the 4% Al isoconcentration surface. **g,** Compositional histograms across the 1.3% Cu isoconcentration surface. All error bars were calculated from counting statistics using the method described in reference [8].

Additional sample of aged Cu-ZSM-5

Data was successfully collected for a second sample of aged Cu-ZSM-5. However, during the analysis of this material it was apparent that it had significant Ga damage, so we chose not to present this data in the main manuscript. The analysis of the material is shown below and demonstrates that the data collected from the other specimen of aged Cu-ZSM-5 is indeed reproducible, mainly the highly heterogeneous Cu and Al distributions. Ion maps for Al, Cu and Ga are shown in Supplementary Figure 35 and it is evident that the tip of the needle contains a region high in Al and Cu, similar to what was found in needle 3. However, the tip of the needle contains a significant amount of Ga, which was found in all needles due to the final FIB milling step. Since it was impossible to exclude the influence of Ga in creating the region rich in Al and Cu, the Ga rich region was removed as discussed in the “Assessing Ga damage” section. In the case of this needle, this volume also contained the Al and Cu rich regions. Based on the discussions in the “Assessing Ga damage” section the Cu and Al agglomeration would not be caused by the FIB milling, but since we did not feel comfortable analyzing the Ga rich volume we chose to exclude this data set. The two views in Supplementary Figure 35, along with the 2D ion maps, show that these regions are close they are not identical. However, even after removal of the Ga rich region, the RDFs for Al and Cu (Supplementary Figure 36) still show significant agglomerations between these species, consistent with the results from needle 3.

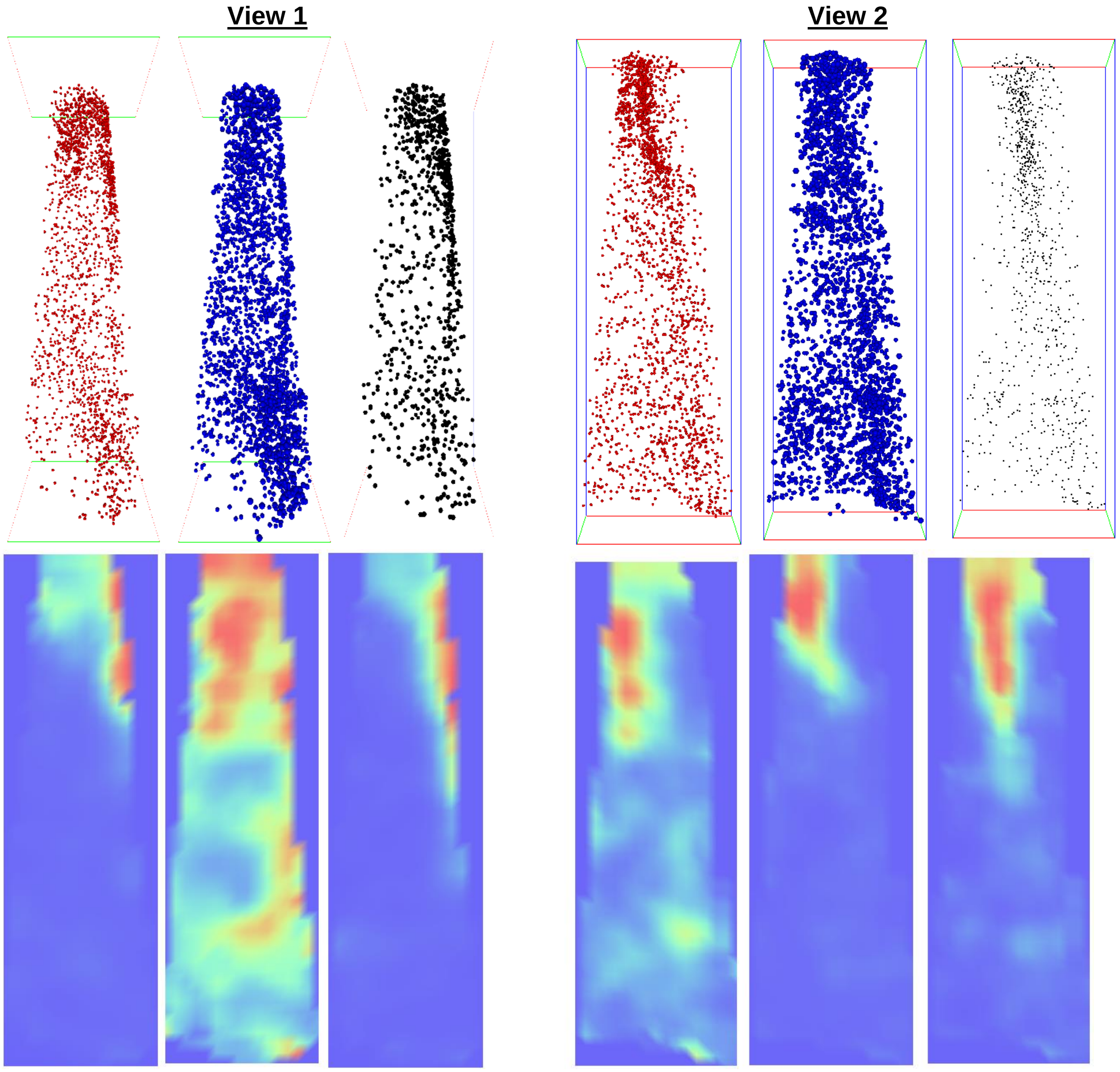

Supplementary Figure 35. Ion maps for Cu (red), Al (blue) and Ga (black) in the second specimen of aged Cu-ZSM-5 which had significant Ga implantation. Views are given down two different axes and the corresponding 2D concentration map is below each ion map. Bounding box dimensions are 50 x 16 x 15 nm^3.

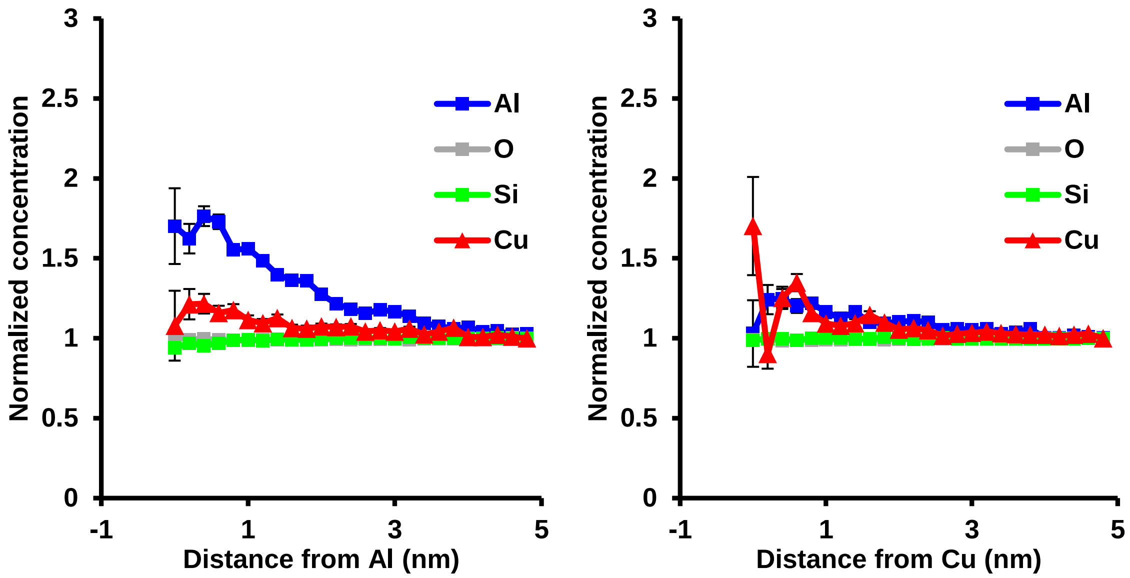

Supplementary Figure 36. Radial distribution function (RDF) for Al (left) and Cu (right) after the removal of the Ga rich portion of the needle. All error bars were calculated from counting statistics using the method described in reference [8].

Supplementary references

1. Giordanino, F. *et al.* Characterization of Cu-exchanged SSZ-13: A comparative FTIR, UV-Vis, and EPR study with Cu-ZSM-5 and Cu-β with similar Si/Al and Cu/Al ratios. *Dalton Trans.* **42,** 12741–61 (2013).
2. Lezcano-Gonzalez, I. *et al.* Chemical deactivation of Cu-SSZ-13 ammonia selective catalytic reduction (NH3-SCR) systems. *Appl. Catal. B Environ.* **154–155,** 339–349 (2014).
3. Lezcano-Gonzalez, I. *et al.* Determining the storage, availability and reactivity of NH3 within Cu-Chabazite-based ammonia selective catalytic reduction systems. *Phys. Chem. Chem. Phys.* **16,** 1639–1650 (2014).
4. Deka, U. *et al.* Confirmation of isolated Cu2+ ions in SSZ-13 zeolite as active sites in NH3-selective catalytic reduction. *J. Phys. Chem. C* **116,** 4809–4818 (2012).
5. Beale, A. M., Gao, F., Lezcano-Gonzalez, I., Peden, C. H. F. & Szanyi, J. Recent advances in automotive catalysis for NOx emission control by small-pore microporous materials. *Chem. Soc. Rev.* **44,** 7371–7405 (2015).
6. Bayal, N. & Jeevanandam, P. Synthesis of metal aluminate nanoparticles by sol-gel method and studies on their reactivity. *J. Alloys Compd.* **516,** 27–32 (2012).
7. Albarracin-Caballero, J. D. *et al.* Structural and kinetic changes to small-pore Cu-zeolites after hydrothermal aging treatments and selective catalytic reduction of NOx with ammonia. *React. Chem. Eng.* **2,** 168–179 (2017).
8. Miller, M. K. *Atom Probe Tomography*. (Springer US, 2000).
9. Larson, D. J., Prosa, T. J., Ulfig, R. M., Geiser, B. P. & Kelly, T. F. *Local Electrode Atom Probe Tomography*. (Springer New York, 2013).
10. Schmidt, J. E. *et al.* Coke Formation in a Zeolite Crystal During the Methanol-to-Hydrocarbons Reaction as Studied with Atom Probe Tomography. *Angew. Chem. Int. Ed.* **55,** 11173–11177 (2016).
11. La Fontaine, A. *et al.* Interpreting atom probe data from chromium oxide scales. *Ultramicroscopy* **159,** 354–359 (2015).
12. Philippe, T. *et al.* Clustering and nearest neighbour distances in atom-probe tomography. *Ultramicroscopy* **109,** 1304–1309 (2009).
13. Sudbrack, C. K., Noebe, R. D. & Seidman, D. N. Direct observations of nucleation in a nondilute multicomponent alloy. *Phys. Rev. B* **73,** 212101 (2006).
14. Haley, D., Petersen, T., Barton, G. & Ringer, S. P. Influence of field evaporation on radial distribution functions in atom probe tomography. *Philos. Mag.* **89,** 925–943 (2009).
15. Miller, M. K. & Forbes, R. G. Atom probe tomography. *Mater. Charact.* **60,** 461–469 (2009).
16. Kelly, T. F. & Miller, M. K. Atom probe tomography. *Rev. Sci. Instrum.* **78,** 31101 (2007).
17. Miller, M. K., Kelly, T. F., Rajan, K. & Ringer, S. P. The future of atom probe tomography. *Mater. Today* **15,** 158–165 (2012).
