# Supplementary material for: Nanoscale tomography reveals the deactivation of automotive copper-exchanged zeolite catalysts
Source: Nat Commun. 2017 Nov 21;8:1666. doi: 10.1038/s41467-017-01765-0 (PMC5698465; doi:10.1038/s41467-017-01765-0)
Supplement: Supplementary file 3 — Description of Additional Supplementary Files [file 41467_2017_1765_MOESM3_ESM.pdf]

List of movies

Supplementary Movie 1. Fresh Cu-ZSM-5, sample 1, 1.8% Cu isoconcentration surface shown with all Al (blue) and Cu (red) ions.
Supplementary Movie 2. Fresh Cu-ZSM-5, sample 2, 2.7% Cu isoconcentration surface shown with all Al (blue) and Cu (red) ions.
Supplementary Movie 3. Aged Cu-ZSM-5, sample 3, 5% Cu and 8% Al isoconcentration surfaces shown with all Al (blue) and Cu (red) ions.
Supplementary Movie 4. Fresh Cu-SSZ-13, Sample 4, all Cu ions.
Supplementary Movie 5. Fresh Cu-SSZ-13, Sample 4, all Cu clusters.
Supplementary Movie 6. Fresh Cu-SSZ-13, Sample 5, all Cu and Al ions.
Supplementary Movie 7. Fresh Cu-SSZ-13, Sample 5, all Cu clusters.
Supplementary Movie 8. Aged Cu-SSZ-13, sample 6, 1.4% Cu isoconcentration surface, shown with all Al ions (blue) and Cu ions (red).
Supplementary Movie 9. Aged Cu-SSZ-13, sample 6, Cu clusters.
Supplementary Movie 10. Aged Cu-SSZ-13, sample 7 1.3% Cu isoconcentration surface and 4% Al isoconcentration surface, shown with all Al ions (blue) and Cu ions (red).
